# The Efficacy of Chinese Herbal Medicine in Animal Models of Polycystic Ovary Syndrome: A Systematic Review and Meta-Analysis

**DOI:** 10.1155/2022/4892215

**Published:** 2022-08-13

**Authors:** Jiacheng Zhang, Xiyan Xin, Haolin Zhang, Yutian Zhu, Yang Ye, Dong Li

**Affiliations:** Department of Traditional Chinese Medicine, Peking University Third Hospital, Beijing 100191, China

## Abstract

**Objective:**

This study aimed to evaluate the efficacy of Chinese herbal medicine (CHM) on ovarian mass, weight, sex hormone disorders, and insulin resistance in animal models of polycystic ovary syndrome (PCOS).

**Methods:**

This systematic review and meta-analysis was conducted through a comprehensive search in three databases to find studies testing CHM in animal models of PCOS. Two researchers independently reviewed the retrieval, extraction, and quality assessment of the dataset. The pooled effects were calculated using random-effect models; heterogeneity was explored through subgroup analysis; and stability was assessed through sensitivity analysis. In addition, publication bias was assessed using the Egger's bias test.

**Results:**

Fifteen studies with twelve mice and 463 rats published from 2016 to 2021 met the inclusion criteria. The results of primary outcomes revealed that CHM therapy was significantly different with control animals in ovarian mass and testosterone (SMD, −1.01 (95% CI, −1.58, −1.45); SMD, −1.62 (95% CI, −2.07, −1.16), respectively). The secondary outcomes as well showed an overall positive effect of CHM compared with control animals in weight (SMD, −1.02 (95% CI, −1.39, −0.65)), follicle-stimulating hormone (FSH) (SMD, 0.58 (95% CI, 0.19, 0.97)), luteinizing hormone (LH) (SMD, −0.94 [95% CI, −1.25, −0.64)), homeostasis model assessment-insulin resistance (HOMA-IR) (SMD, −1.24 (95% CI, −1.57, −0.92)). Subgroup analyses indicated that PCOS induction drug, formula composition, random allocation, and assessment of model establishment were relevant factors that influenced the effects of interventions. The stability of the meta-analysis was showed robust through sensitivity analysis. The publication bias was substantial.

**Conclusions:**

Administration with CHM revealed a statistically positive effect on ovarian mass, weight, sex hormone disorders, and insulin resistance. Moreover, these data call for further high-quality studies investigating the underlying mechanism in more depth.

## 1. Introduction

Polycystic ovary syndrome (PCOS), characterized mainly by hyperandrogenism (androgen excess, acne and/or hirsutism) and ovarian dysfunction (failure or absence of ovulation and/or polycystic ovarian morphology), is one of the most common endocrine and metabolic disorders. It affects approximately 8% to 18% of women of reproductive age [1]. However, most of the drugs treating the symptoms of PCOS were used in an off-label fashion because no drug was approved specifically for PCOS neither by the FDA nor by the European Medicines Agency [2]. The first-line therapy for PCOS is lifestyle modification via diet and physical activity [3]. Patients with mild symptoms might not require any drug intervention. Besides, letrozole and clomiphene are considered the most common drugs recommended for patients with PCOS who are seeking fertility [4]. According to a consensus statement, the oral contraceptive pill and antiandrogens are suggested in PCOS patients who are not attempting to conceive [5]. Notwithstanding their efficacy, clinical resistance, multiple pregnancy along with ovarian hyperstimulation syndrome are prone to occur with chronic treatment [6]. Considering that PCOS is a heterogeneous and lifelong disorder, treatment for PCOS should be symptom-oriented and adapted to the expectations of the individual patient.

Traditional Chinese medicine (TCM), attaching great importance to individualized treatment, follows an independent theoretical pathway to make diagnosis and treatment plans by systematically evaluating patients' signs and symptoms. Gynaecological and infertility problems of PCOS have been widely treated by TCM in China [7, 8]. Some women in southern Australia, the UK, and the USA have also begun to use TCM in subfertility clinics [9]. Chinese herbal medicine (CHM), a kind of TCM therapy consisting of a variety of herbs, remains controversial because it is hard to be standardized. CHM has different compatibility for each patient which coincides with the principle that we should pay attention to individualized treatment in PCOS. Notwithstanding the growing interest in CHM, rigorous clinical trials addressing their specific effects are lacking. These trials showed quite a few biases related to age, genetic background, or other interfering variables [10]. In this regard, animal models may represent a useful tool to study the efficacy and mechanisms of various formulas used in PCOS models that were induced by letrozole, dehydroepiandrosterone, prasterone sodium sulfate, or testosterone propionate. In addition, rats and mice are ideal animal models for PCOS because they are sensitive to sexual hormone stimulation and have a stable estrous cycle that is easy to observe [11, 12].

The components of different formulas act synergistically in various ways [13]. Our previous study demonstrated that Bushen Huatan Granules (BHG) and Kunling Wan (KW) respectively ameliorated DHEA-induced PCOS symptoms such as irregular estrous cycle, high levels of testosterone and insulin in serum. Both BHG and KW may attenuate the apoptosis in granulosa cells (GCs), while BHG targets the mitochondria-dependent apoptotic pathway and KW targets the endoplasmic reticulum stress-dependent apoptotic pathway [14]. In addition, it is reported that Guizhi Fuling Wan (GFW) inhibited GCs' autophagy by activating the phosphatidylinositol-3-Kinase (PI3K)/Protein Kinase B (AKT)/mammalian Target of Rapamycin (mTOR) pathway and alleviated ovulation disorder in PCOS-IR rats [15]. Considering the optimal pattern of CHM therapy in various symptoms of PCOS remains unanswered, analysis that evaluates the specific efficacy of different formulas in rodent models of PCOS will be prospective.

Herein, we report a systematic review and meta-analysis of data from studies testing the efficacy of CHM in animal models of PCOS. The purpose of this study was to provide evidence relating to the efficacy of CHM on ovarian mass, weight, sex hormone disorders, and insulin resistance in animal models of PCOS.

## 2. Methods

We conducted the systematic review in accordance with the Preferred Reporting Items for Systematic Reviews and Meta-Analysis (PRISMA) guidelines [16, 17]. No animal work was performed in this study. Moreover, the protocol was based on SYRCLE's systematic review protocol format for animal intervention [18] was registered in PROSPERO (registration number: CRD42022310345).

### 2.1. Search Strategy

We conducted a comprehensive search of three electronic databases which included PubMed, Web of Science, and Scopus from inception to February 2022. Searches were limited to English-language publications. Details of the search strategies are shown in Supplementary File 1.

### 2.2. Inclusion and Exclusion Criteria

The experts formulated the eligibility criteria for the current study. Publications were considered eligible based on the following inclusion criteria: (1) Experimental subjects are animal models of PCOS. (2) Intervention is Chinese herbal medicine. (3) Comparison with the PCOS group in the animal experimental studies, with no treatment. (4) Outcomes include effects of Chinese herbal medicine on the development or treatment of PCOS, morphological and hormonal alterations in the animal experimental studies. The original trials should include at least one of the following outcomes: weight, ovarian mass, follicle-stimulating hormone (FSH), luteinizing hormone (LH), testosterone, and homeostasis model assessment-insulin resistance (HOMA-IR). (5) Types of study are experimental animal studies.

Furthermore, two authors examined the titles and abstracts of retrieved studies. Exclusion criteria were: (1) nonoriginal studies and no full-text articles (e.g. reviews, editorials); (2) in vitro and in silico studies; (3) interventions different from Chinese herbal medicines (e.g. single traditional Chinese medicine, acupuncture or monomer composition); (4) all species different from mouse and rat; (5) presence of concomitant interventions in the control group. In addition, full-texts of retrieved studies were assessed for eligibility independently by two authors. Studies for which was no full-text available or had no relevant outcomes reported were excluded.

### 2.3. Data Extraction

Two reviewers independently assessed the extraction of data. Any disagreement between them was solved through discussion. Detailed data extraction was collected by using the following characteristics: (1) publication details (author and year); (2) intervention used (prescription composition, route, dose and timing); (3) PCOS induction method; (4) animal used (species, strain, age and weight); (5) underlying mechanism of the intervention; (6) information of outcomes. Moreover, for each comparison, we extracted data reporting the sample size per group, mean value, and variance (SD or SEM) for both the treatment and control group.

The sample size of the control group was divided by the number of treatment groups to adjust the impact of the control group [19]. When treatment was administered in multiple doses, each dose's data was extracted and performed in separate studies. For studies only presented graphically, we contacted the corresponding authors by e-mail to request data. If no response was received, we used the ImageJ software to quantify the results. We will exclude studies when essential data could not be obtained.

### 2.4. Risk of Bias Assessment

The internal validity of the included studies was assessed by two reviewers independently, referencing the SYRCLE's risk of bias tool for animal studies [20]. The 10-item checklist of evaluation included: (1) publication in a peer-reviewed journal; (2) control of temperature; (3) random allocation to groups; (4) assessment of PCOS model establishment; (5) blinded assessment of outcome; (6) accurate drug production institutions; (7) detection of estrous cycles; (8) the use of comorbidity animals; (9) compliance with animal welfare regulations; 10) statement of potential conflicts of interest.

### 2.5. Statistical Analysis

When the measure of variance reported was Standard Error of Mean (SEM), we inverted SEM to Standard Deviation (SD) first. Considering that different species and measurements in animals vary greatly, we used a standardized mean difference (SMD) to standardize the results to a uniform scale [21].

We performed a random-effect meta-analysis to calculate the SMD values with 95% confidence intervals (CI) as the overall effects for the combined pooled outcomes which were continuous. Heterogeneity was assessed by the *Q* statistic and quantified using the *I*^2^ statistic [22]. Further, subgroup analyses, related to the impacts of interventions with different mechanisms and characteristics, were conducted to explore the sources of heterogeneity. Sensitivity analyses were performed by repeating the primary meta-analysis to confirm the robustness of the results. Moreover, publication bias was assessed statistically with Egger's bias test with *p* < 0.05 indicating asymmetry. RevMan 5.3 and STATA 17 were used to perform the statistical analyses.

## 3. Results

### 3.1. Study Selection

**Figure 1:**
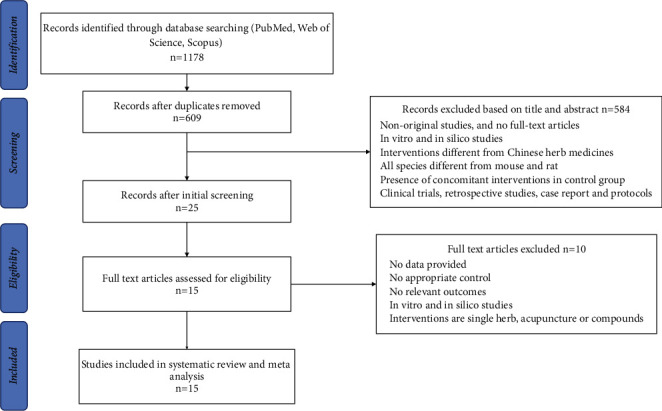
Flow diagram of publication inclusion.

Initially, 1178 studies were retrieved through a comprehensive search of three databases (66 for PubMed, 1069 for Web of Science, and 43 for Scopus), out of which 609 nonduplicate studies were filtered out. After reviewing the titles and abstracts, 584 studies were removed based on predetermined exclusion criteria. Furthermore, 10 studies were excluded in the second selection phase, and 15 studies were finally included in the systematic review. A flowchart depicting the research selection process is presented in Figure 1.

### 3.2. Study Characteristics

These 15 articles [14, 15, 23–35] investigated 13 formulas and 35 treatment arms according to dose and herb composition. The main characteristics are summarized in Table 1. All the included studies were conducted between 2016 and 2021. Rats were used in most of the studies, only one study used mice. The quantity of herbal medicine used in compounds varied greatly, which ranged from 2 to 31. PCOS models were induced by letrozole, dehydroepiandrosterone (DHEA), prastenone sodium sulfate, or testosterone propionate (TP). Moreover, the characteristics of the administration route differed sketchily among the studies. Nine studies administered the CHM by gavage, three indicated the oral route without specifications, and three studies did not mention the administration route. With regard to the outcomes of interest to us, the 15 articles contained 13 comparisons for ovarian mass and 31 comparisons for testosterone as the primary outcomes. Weight (in 19 comparisons), FSH (in 20 comparisons), LH (in 21 comparisons), and HOMA-IR (in 18 comparisons) were assessed as the secondary outcomes.

### 3.3. Study Quality

**Figure 2:**
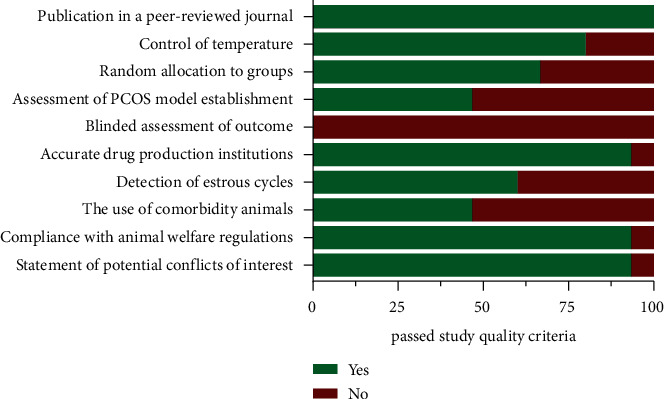
Study quality assessment of the included publications.

The median study quality score was 7 of a possible 10 (interquartile range, 5 to 8). All studies included were published in peer-reviewed journals. Twelve articles (80%) reported control of temperature during study. Ten articles (67%) indicated random allocation. The criteria assessing PCOS model establishment were reported in seven articles (47%). No article reported a blinded assessment of the outcome, and only one publication did not report the accurate drug production institutions. Nine articles (60%) detected estrous cycles before the experiment. Relevant comorbidity was modeled in seven studies (47%). Besides, only one article did not report compliance with animal welfare and a conflicts of interest statement.  Figure 2 and Supplementary Table 1 provide detailed information about each study quality.

### 3.4. Primary Outcomes

#### 3.4.1. Ovarian Mass

**Figure 3:**
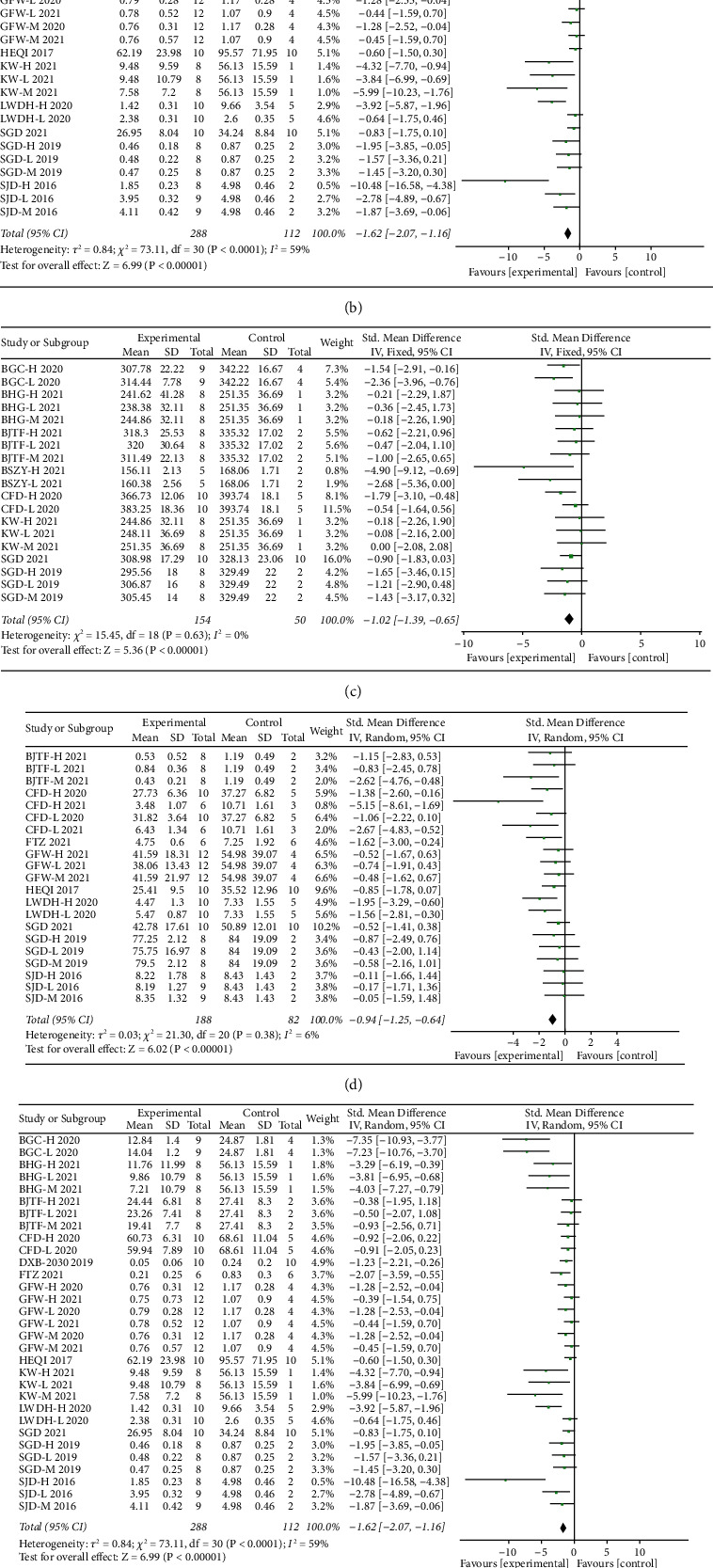
Forest plots of outcomes. (a) Ovarian mass; (b) testosterone; (c) weight; (d) FSH; (e) LH; (f) HOMA-IR.

Six studies, including 13 comparisons measured the efficacy of CHM in PCOS. Overall, the random-effect model showed that administration of CHM led to a significant decrease of ovarian mass in animal models of PCOS (SMD = −1.01, 95% CI: −1.58 to −0.45). Heterogeneity between studies was substantial (*I*^2^ = 52%) (Figure 3(a)).

#### 3.4.2. Testosterone

Thirteen studies, including 31 comparisons reported testosterone. The random-effect model showed that CHM therapy was associated with a significant difference compared with the control group (SMD = −1.62, 95% CI: −2.07 to −1.16). Heterogeneity between studies was substantial (*I*^2^ = 59%) (Figure 3(b)).

### 3.5. Secondary Outcomes

#### 3.5.1. Weight

Seven studies, including 19 comparisons reported weight. The random-effect model showed that CHM therapy was associated with a significant difference compared with the control group (SMD = −1.02, 95% CI: −1.39 to −0.65) (Figure 3(c)).

#### 3.5.2. FSH

Nine studies, including 20 comparisons reported FSH. The random-effect model showed that CHM therapy was associated with a significant difference compared with the control group (SMD = 0.58, 95% CI: 0.19 to 0.97) (Figure 3(d)).

#### 3.5.3. LH

Ten studies, including 21 comparisons reported LH. The random-effect model showed that CHM therapy was associated with a significant difference compared with the control group (SMD = −0.94, 95% CI: −1.25 to −0.64) (Figure 3(e)).

#### 3.5.4. HOMA-IR

Seven studies, including 18 comparisons reported HOMA-IR. The random-effect model showed that CHM therapy was associated with a significant difference compared with the control group (SMD = −1.24, 95% CI: −1.57 to −0.92) (Figure 3(f)).

### 3.6. Subgroup Analyses

The heterogeneity of the secondary outcomes which included weight, FSH, LH, and HOMA-IR is little (*I*^2^ = 0%; *I*^2^ = 40%; *I*^2^ = 6%; *I*^2^ = 0%, respectively). The pooled estimates for studies in meta-analyses of ovarian mass and testosterone exhibited substantial heterogeneity (*I*^2^ = 52%; *I*^2^ = 59%). Subgroup analyses were conducted to identify the source of heterogeneity with five covariates (PCOS induction drug, formula composition, control of temperature, random allocation, and assessment of model establishment).

For ovarian mass, administration of formulas with different compositions may be a possible source of heterogeneity (*P*=0.003) (Supplementary Figure 1B). Besides, treatment effects were higher in studies that reported random allocation (*P*=0.02) (Supplementary Figure 1D) and in studies that reported assessment of model establishment (*P*=0.05) (Supplementary Figure 1E). Overall, analyses showed that PCOS induction drugs and control of temperature did not account for the heterogeneity and different estimates of efficacy for ovarian mass (*P*=0.44; *P*=0.47) (Supplementary Figures 1A/1C).

For testosterone, we found significant impact of different formulas used in the PCOS model (*P* < 0.01) (Supplementary Figure 2B). Analysis showed that PCOS induction drug may account for a proportion of the heterogeneity of the efficacy for testosterone (*P*=0.07) (Supplementary Figure 2A). The other covariates including control of temperature, random allocation, and assessment of model establishment were shown to be irrelevant to the heterogeneity (*P*=0.7; *P*=0.2; *P*=0.28, respectively) (Supplementary Figures 2C/2D/2E).

**Figure 4:**
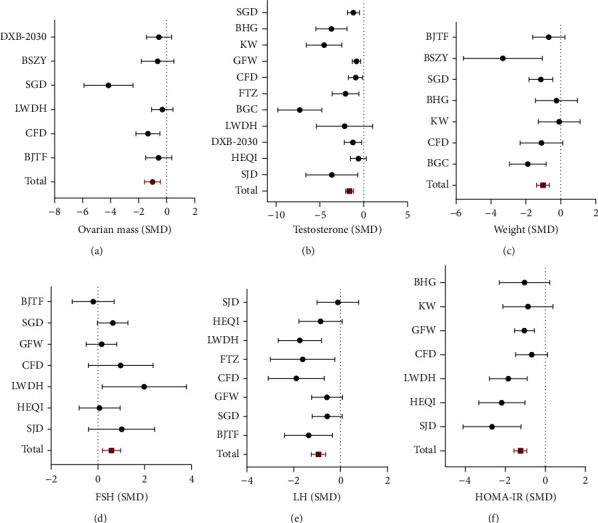
Forest plots of efficacy of different formulas on PCOS in animal models. (a) Ovarian mass; (b) testosterone; (c) weight; (d) FSH; (e) LH; (f) HOMA-IR.

Furthermore, considering that various formulas have different emphasis on the improvement effects, subgroup analyses of formula composition for the other outcomes were conducted. Analyses showed that in concluded studies the effect of Shaoyao-Gancao Decoction (SGD) on reducing ovarian mass was the best (SMD = −4.16, 95% CI: −5.91 to −2.4), the effect of Bao Gui capsule (BGC) on reducing testosterone was the best (SMD = −7.29, 95% CI: −9.8 to −4.77), the effect of Bu-Shen-Zhu-Yun Decoction (BSZY) on reducing weight was the best (SMD = −3.32, 95% CI: −5.58 to −1.06), the effect of Liuwei Dihuang Pills (LWDH) on improving FSH was the best (SMD = 1.98, 95% CI: 0.18 to 3.79), the effect of Cangfudaotan Decoction (CFD) on reducing LH was the best (SMD = −1.89, 95% CI: −3.09 to −0.69), and the effect of Shouwu Jiangqi Decoction (SJD) on reducing HOMA-IR was the best (SMD = −2.66, 95% CI: −4.11 to −1.22) (Figure 4 and Supplementary Figure 3).

### 3.7. Sensitivity Analyses

The sensitivity analysis was performed by removing one study at a time to confirm and account for the stability of the results. The pooled sensitivity analyses showed that the results would not be affected by excluding any study, which suggested that the stability of the results remained robust (Supplementary Figure 4).

### 3.8. Publication Bias

**Figure 5:**
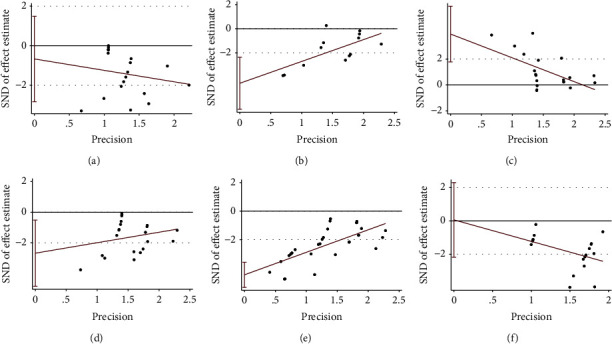
Publication bias assessment. Egger's test for (a) weight; (b) ovarian mass; (c) FSH; (d) LH; (e) testosterone; (f) HOMA-IR.

The Egger's bias test was performed to identify the potential publication bias. For ovarian mass, FSH and testosterone, significant publication bias was detected (*P* < 0.01) (Figures 5(b), 5(c) and 5(e)).

## 4. Discussion

In this study, we performed a meta-analysis aimed at assessing the efficacy of CHM therapy in animal models of PCOS. Compared with other publications, the strengths of this study consisted of only animal experiments being included for the meta-analysis which differed from the analyses for comparing randomized controlled trials (RCTs) [10, 36], different formulas and doses being compared to verify the most appropriate treatment, and multivariate subgroup analyses of square law and experimental design. Overall, results of the study indicated that some specific features of PCOS (ovarian mass, weight, sex hormone disorders, and insulin resistance) were ameliorated by CHM. Specifically, the ovarian mass, testosterone, weight, LH, and HOMA-IR were decreased, and the FSH was increased. The outcomes of ovarian mass and testosterone represented substantial heterogeneity, and their sources mainly were formula composition, random allocation, assessment of model establishment, and PCOS induction drug. According to the subgroup analyses, different formulas showed different emphasis on the improvement effects on various symptoms of PCOS. Besides, the Egger's bias test showed substantial publication bias in the outcomes of ovarian mass, FSH, and testosterone.

Overall, the study quality of the studies included was moderate, and there was little difference in the quality of articles. We tried to perform a correlation analysis between the quality and the year of the study, but no statistical correlation was found (data not shown). Among the 15 studies, 10 publications reported the random allocation, and no publication mentioned the blinded assessment of outcomes, which implied that in the animal models blinding methods are usually seen as technical difficulties. Given that failure to perform blind assessment might lead to overestimation of effect sizes, we recommend following more standardized experimental standards in preclinical studies. Besides, contrary to our expectations, randomization to group was associated with a higher improvement in outcomes of testosterone. We speculate that expected results may be anticipated if they follow strict experimental criteria.

Seven publications reported the assessment of the PCOS model establishment. In our study, the best effect was seen in the publication that evaluated the PCOS model establishment before intervention. This was probably because the improvement of phenotype by CHM was more remarkable in the exact model. 7 publications employed comorbidity animals with obesity, hyperglycemia, or chronic stress states which may be more in line with the pathophysiology of PCOS patients [37, 38].

It has been estimated that as many as 38% to 88% of women with PCOS are overweight or obese, but that does not mean obesity is a defining feature of PCOS as the syndrome is also seen in women of normal weight [39]. Hyperandrogen, metabolic dysfunction, and insulin resistance caused by PCOS are the significant predisposition of weight gain [40]. The subgroup analyses exploring the source of heterogeneity of ovarian mass and weight revealed that better effectiveness was shown in letrozole-induced models (Supplementary Figure 1A/Figure 5). Among the studies included, 9 publications used letrozole, 3 publications used prasterone sulfate sodium, and only one publication used testosterone propionate. As a nonsteroidal aromatase inhibitor, letrozole restrains the conversion of androgen to estrogen, leading to androgen accumulation and ultimately recapitulating both reproductive and metabolic PCOS phenotypes in rats and mice [41, 42]. The PCOS models induced by testosterone propionate were characterized by high blood free testosterone level, low LH and FSH values, which can last for a long time [43, 44]. As another androgen induction method, DHEA and prasterone sulfate sodium require shorter modeling time than testosterone propionate and are closer to the pathogenesis of PCOS [45, 46]. In addition to the modeling methods mentioned in this study, estradiol valerate, insulin combined with human chorionic gonadotrophin (HCG), and progesterone combined with HCG have also been used in other studies to induce PCOS models [47–49]. There were also studies modeling PCOS through torsion-detorsion and found that apoptosis caused by oxidative stress is an important factor in ovarian tissue damage [50]. Afterwards, they found that *Galega officinalis* extract and coenzyme Q10 could reverse this pathological change [51]. As PCOS is a highly heterogeneous disease, an animal model that can fully simulate the clinical characteristics of PCOS is not realistic. We suggest that appropriate modeling methods should be selected according to the purpose of the study.

Systematic review of preclinical studies is conducive to exploring the potential mechanism of CHM in ameliorating symptoms of PCOS. Our study included 2 publications with SGD as the intervention. The efficacy of SGD on decreasing ovarian mass was the best. SGD might reduce the phosphorylation of nuclear factor kappa-B (NF-kB) p65, suppress toll like receptor 4 (TLR4)/NF-kB signaling pathway, remodel gut microbiome structure, and protect gut barrier, which leads to ameliorate the inflammatory response in the ovary of PCOS rats [25, 33]. As the most effective formula to reduce high testosterone, BGC decreased the expression of P450c17*α* and increased the expression of P450arom to improve hyperandrogenism [30]. There were also studies on the effect of CHM on ovarian granule cell apoptosis through the PKR-like Reticulum Kinase (PERK)-ATK4- C/EBP homologous protein (CHOP) signaling pathway, PI3K/AKT/mTOR pathway, and P13K/AKT/Forkhead Box Protein O1A (FOXO1A) pathway [15, 23, 24, 31]. Furthermore, studies on the mechanism of CHM improving PCOS insulin resistance generally explored the glucose transporter 4 (GLU4) related pathways [27, 32].

PCOS is a very complex disease in the human body. Animal experiments can simplify the complex pathological process, and subtle research on various inducing factors can be carried out to recommend the best effect of the CHM formula on PCOS patients with different pathogeny characteristics in clinic. Compared with clinical trials, researchers can more accurately control the dose and method of administration in animal research, without considering the patient's compliance and other difficult problems in clinic. In the meanwhile, animal research is convenient to obtain materials, and the expression of molecular markers can be easily evaluated. This enabled our systematic review to summarize the mechanism of CHM in ameliorating symptoms of PCOS. Besides, our results pointed out that the efficacy of CHM prescriptions on various models was different, which indicated that the clinical application should also give attention to the advantages of individualized treatment of CHM.

The pooled sensitivity analyses showed the stability of the results. The Egger's bias test suggested the substantial presence of publication bias in ovarian mass, FSH, and testosterone, which indicated that the efficacy might be overestimated. Only English-language studies being included may account for the publication bias. Our initial plan was to include at least 10 articles in each result, but the reality is that some important outcomes were not assessed in the included articles. Even though the number of articles in some results did not meet the protocol, we still conducted the data integration. Our study also has the following limitations: First, the publications included were searched in English-language databases, so the databases in other languages were excluded. Moreover, gray literature and negative results were also lacking. Second, the number of studies assessing the pregnancy rate and litter size is too limited to conduct the meta-analysis, which means the effect of CHM on infertility in animal models could not be evaluated. Third, only one study used the mice model, so we could not conduct the subgroup analysis by species. Finally, although we tried to analyze more outcomes, the limited quantity of included studies made it unpractical.

## 5. Conclusion

To the best of our knowledge, this is the first systematic review and meta-analysis that evaluated the efficacy of CHM on PCOS in animal models. We concluded that CHM resulted in improvements in ovarian mass, weight, FSH, LH, testosterone, and HOMA-IR in animal models of PCOS. The improvement effects of different formulas are targeted. For instance, SGD reduced ovarian mass the most, and BGC decreased testosterone levels the most. Furthermore, we suggest using PCOS modeling drugs that meet the research purpose when studying different mechanisms. Overall, the results should be interpreted with caution because of substantial heterogeneity and publication bias.

## Figures and Tables

**Table 1:** Characteristics of included studies.

Author, year	Intervention	Mechanism	Quantity of herb types	Dose(s)	Route	Treatment duration	PCOS induction methods	Species	Strain	Outcomes
Pan, 2021	BJTF	PERK-ATF4-CHOP↓	8	BJTF-L: 9.32 g/kg	Gavage	21 days	Daily gavage of letrozole, 1 mg/kg, for 28 days	Rat	SD	①②③④⑤
				BJTF-M: 18.63 g/kg						
				BJTF-H: 37.26 g/kg						

Jiang, 2021	BSZY	PI3K/AKT/mTOR↑	6	BSZY-L: 20 g/kg	Oral	6 estrous cycles	Daily s.c injection of DHEA, 60 mg/kg, for 28 days	Rat	SD	①③
				BSZY-H: 40 g/kg						

Chang, 2021	SGD	TLR4/NF-*κ*B↓	2	25 g/kg	NM	2 weeks	Daily oral administration of letrozole, 1 mg/kg, for 21 days	Rat	SD	②③③⑤

Xu, 2021	BHG; KW	BHG: regulation of mitochondria	BHG: 10	BHG-L: 0.75 g/kg	Gavage	28 days	Daily s.c injection of DHEA, 60 mg/kg, for 20 days	Rat	SD	②③⑥
				BHG-M: 1.49 g/kg						
				BHG-H: 2.99 g/kg						
		KW: protection of endoplasmic reticulum stress.	KW: 31	KW-L: 0.46 g/kg						
				KW-M: 0.91 g/kg						
				KW-H: 1.82 g/kg						

Liu, 2021	GFW	PI3K/AKT/mTOR↑	5	GFW-L: 0.31 g/kg	Gavage	30 days	Daily oral administration of letrozole combined with intragastric high-fat emulsion, for 21 days	Rat	SD	②④⑤⑥
				GFW-M: 0.62 g/kg						
				GFW-H: 1.24 g/kg						

Wang, 2020	CFD	IGF-1-PI3K/Akt-Bax/Bcl-2↓	16	CFD-L: 15 g/kg	Gavage	4 weeks	Daily intraperitoneal injection of letrozole, 1 mg/kg, combined with HFD, for 21 days	Rat	SD	①②③④⑤⑥
				CFD-H: 30 g/kg						

Xu, 2021	FTZ	Release of adiponectin↑	8	2.892 g/kg	Gavage	5 weeks	Daily intraperitoneal injection of letrozole, 50 *μ*g/d, combined with HFD, for 35 days	Mouse	C57 BL/6J	②⑤

Yi, 2021	CFD	OATP2B1 and OATP3A1↑	9	CFD-L: 1.42 g/kg	Gavage	2 weeks	Daily s.c injection of prasterone sodium sulfate, 90 mg/kg, combined with Kcal fat diet, for 40 days	Rat	SD	④⑤
				CFD-H: 5.68 g/kg						

Zhu, 2020	GFW	Reshape the intestinal flora	5	GFW-L: 0.31 g/kg	Gavage	35 days	Daily gavage of letrozole, 1 mg/kg, combined with intragastric high-fat emulsion, for 35 days	Rat	SD	②⑥
				GFW-M: 0.62 g/kg						
				GFW-H: 1.24 g/kg						

Lian, 2020	BGC	P450c17*α*↓, P450arom↑, GLUT4↑	12	BGC-L: 0.28 g/kg	Gavage	3 weeks	Daily gavage of letrozole, 1 mg/kg, for 21 days	Rat	SD	②③
				BGC-H: 0.57 g/kg						

Qiu, 2020	LWDH	PI3K/AKT/FOXO1A↑	6	LWDH-L: 1.2 g/kg	NM	21 days	Daily gavage of letrozole, 1 mg/kg, combined with HFD, for 21 days	Rat	SD	①②④⑤⑥
				LWDH-H: 3.6 g/kg						

Azeemuddin, 2019	DXB-2030	GLUT4↑	5	100 mg/kg	NM	60 days	S.c injection of TP, 1.25 mg/pup, for 70 days.	Rat	Wistar	①②

Shao, 2019	SGD	NF-*κ*B↓	2	SGD-L: 12.5 g/kg	Oral	2 weeks	Daily oral administration of letrozole, 1 mg/kg	Rat	NM	①②③④⑤
				SGD-M: 25 g/kg						
				SGD-H: 50 g/kg						

Zhao, 2017	HEQI	PI3K/AKT↑	12	8.1 g/kg	Oral	30 days	Daily s.c injection of DHEA, 60 mg/kg, for 20 days	Rat	SD	②④⑤⑥

Wang, 2016	SJD	IRS-1↑, PI3K p85*α*↑	6	SJD-L: 9.2 g/kg	Gavage	18 days	Daily s.c injection of prasterone sodium sulfate, 90 mg/kg, combined with HFD, for 42 days	Rat	SD	②④⑤⑥
				SJD-M: 18.4 g/kg						
				SJD-H: 36.8 g/kg						

Interventions. BGC, bao gui capsule; BHG, bushen huatan granules; BJTF, bushen jieyu tiaochong formula; BSZY, bu-shen-zhu-yun decoction; CFD, cangfudaotan decoction; FTZ: fu fang zhenzhu tiaozi; GFW, guizhi fuling wan; HEQI, heqi san; KW, kunling wan; LWDH, liuwei dihuang pills; SGD, shaoyao-gancao decoction; SJD, shouwu jiangqi decoction. Mechanism, ATF4: activation transcription factor 4; AKT, protein kinase B; Bax, BCL2-associated X; Bcl-2, B-cell lymphoma-2; CHOP, C/EBP homologous protein; FOXO1A, forkhead box protein O1A; IGF-1, insulin-like growth factor-1; IRS-1, insulin receptor substrate-1; mTOR, mammalian target of rapamycin; NF-*κ*B, nuclear factor kappa-B; OATP2B1, organic anion transporting polypeptide 2b1; OATP3A1, organic anion transporting polypeptide 3a1; PERK, PKR-like reticulum kinase; PI3K: phosphatidylinositol-3-kinase; P450arom, P450 aromatase; P450c17*α*, cytochrome P450, family 17, subfamily A, polypeptide 1; TLR4: toll like receptor 4. Route: NM: not mentioned. PCOS induction methods: DHEA: dehydroepiandrosterone; HFD: high-fat diet; S.c, subcutaneous; TP, testosterone propionate. Strain: SD, Sprague–Dawley. Outcome measurements: ① ovarian mass; ② testosterone; ③ weight; ④ FSH; ⑤ LH; ⑥ HOMA-IR.

## Data Availability

The data used to support the findings of this study are available from the corresponding author upon request.

## Supplementary Materials

Supplementary Materials Supplementary File 1: Search strategy. Table S1: Study quality score report. Figure S1: Forest plot of the efficacy of CHM on ovarian mass. Figure S2: Forest plot of the efficacy of CHM on testosterone. Figure S3: Forest plots of the efficacy of different formulas. Figure S4: Sensitivity analysis. Figure S5: Forest plots of weight improvement by different PCOS induction drugs.
