# Supplementary material for: The Efficacy of Chinese Herbal Medicine in Animal Models of Polycystic Ovary Syndrome: A Systematic Review and Meta-Analysis
Source: Evid Based Complement Alternat Med. 2022 Aug 13;2022:4892215. doi: 10.1155/2022/4892215 (PMC9392647; doi:10.1155/2022/4892215)

SUPPLEMENTAL MATERIAL

Title: The Efficacy of Chinese Herbal Medicine in Animal Models of Polycystic Ovary Syndrome: A Systematic Review and Meta-Analysis

Authors: Jiangcheng Zhang, Xiyan Xin, Haolin Zhang, Yutian Zhu, Yang Ye, Dong Li

Affiliation: Department of Traditional Chinese Medicine, Peking University Third Hospital, 38 Xueyuan Road, Beijing 100191, China

Supplementary File 1. Search strategy

1. Search strategy used in PubMed database.

#1. rat[Title/Abstract] OR rats[Title/Abstract] OR mouse[Title/Abstract] OR mice[Title/Abstract] OR animal[Title/Abstract] OR animals[Title/Abstract] Items found: 3410081

#2. polycystic ovarian syndrome[Title/Abstract] OR polycystic ovary syndrome[Title/Abstract] OR polycystic ovary[Title/Abstract] OR pcos[Title/Abstract] Items found: 18495

#3. TCM[Title/Abstract] OR herbal medicine[Title/Abstract] OR herb[Title/Abstract] OR formula[Title/Abstract] OR decoction[Title/Abstract] OR chinese[Title/Abstract] OR pill[Title/Abstract] OR tang[Title/Abstract] OR granule[Title/Abstract] Items found: 420728

#4. #1 AND #2 AND #3 Items found: 66

2. Search strategy used in Web of Science database

#1. TS=(polycystic ovarian syndrome OR polycystic ovary syndrome OR polycystic ovary OR pcos) Items found: 39588

#2. TS=(rat OR rats OR mouse OR mice OR animal OR animals) Items found: 23088465

#3. TS=(TCM OR herbal medicine OR herb OR formula OR decoction OR chinese OR pill OR tang OR granule) Items found: 3337417

#4. #1 AND #2 AND #3 Items found: 1069

3. Search strategy used in Scopus

#1. TITLE-ABS-KEY (rat OR rats OR mouse OR mice OR animal OR animals) Items found: 8304654

#2. TITLE-ABS-KEY (polycystic AND ovarian AND syndrome OR polycystic AND ovary AND syndrome OR polycystic AND ovary OR pcos) Items found: 11507

#3. TITLE-ABS-KEY (tcm OR herbal AND medicine OR herb OR formula OR decoction OR chinese OR pill OR tang OR granule) Items found: 106434

$4. #1 AND #2 AND #3 Items found: 43

Supplementary Table 1. Study quality score report

Author	Year	(1)	(2)	(3)	(4)	(5)	(6)	(7)	(8)	(9)	(10)	Aggregate quality score
Pan	2021	1	1	0	1	0	1	1	1	1	1	8
Jiang	2021	1	0	1	1	0	1	1	0	1	1	7
Chang	2021	1	1	1	0	0	1	1	0	1	1	7
Xu	2021	1	1	0	0	0	1	1	0	1	1	6
Liu	2021	1	1	1	0	0	1	0	0	1	1	6
Wang	2020	1	1	1	1	0	1	1	0	1	1	8
Xu	2021	1	1	0	1	0	0	1	1	1	1	7
Yi	2021	1	1	1	0	0	1	0	1	1	1	7
Zhu	2020	1	0	1	0	0	1	0	1	1	1	6
Lian	2020	1	1	1	1	0	1	0	0	1	1	7
Qiu	2020	1	1	0	0	0	1	1	1	1	1	7
Azeemuddin	2019	1	1	0	0	0	1	1	1	1	1	7
Shao	2019	1	1	1	1	0	1	1	0	1	1	8
Zhao	2017	1	1	1	0	0	1	0	0	1	1	6
Wang	2016	1	0	1	1	0	1	0	1	0	0	5

(1) Publication in a peer-reviewed journal
(2) Control of temperature
(3) Random allocation to groups
(4) Assessment of PCOS model establishment
(5) Blinded assessment of outcome
(6) Accurate drug production institutions
(7) Detection of estrous cycles
(8) The use of co-morbid animals
(9) Compliance with animal welfare regulations
(10) Statement of potential conflicts of interest

Supplementary Fig. 1 Forest plot of the efficacy of CHM on ovarian mass

A

	Experimental			Control				Std. Mean Difference
Study or Subgroup	Mean	SD	Total	Mean	SD	Total	Weight	IV, Random, 95% CI
**1.1.1 Letrozole**								
BJTF-H 2021	61.5	10.16	8	71.12	10.16	2	6.9%	-0.86 [-2.48, 0.77]
BJTF-L 2021	74.33	16.04	8	71.12	10.16	2	7.3%	0.19 [-1.37, 1.74]
BJTF-M 2021	57.75	10.16	8	71.12	10.16	2	6.6%	-1.19 [-2.88, 0.50]
CFD-H 2020	117.5	26.96	10	157.09	17.61	5	9.1%	-1.52 [-2.77, -0.28]
CFD-L 2020	139.55	11.99	10	157.09	17.61	5	9.5%	-1.18 [-2.36, -0.00]
LWDH-H 2020	133.68	29.02	10	145.08	21.76	5	10.1%	-0.40 [-1.48, 0.69]
LWDH-L 2020	139.9	21.76	10	145.08	21.76	5	10.2%	-0.22 [-1.30, 0.85]
SGD-H 2019	71.36	6.55	8	109.95	3.64	2	2.3%	-5.57 [-9.06, -2.07]
SGD-L 2019	91.75	5.83	8	109.95	3.64	2	4.5%	-2.93 [-5.20, -0.67]
SGD-M 2019	80.83	5.1	8	109.95	3.64	2	2.4%	-5.32 [-8.70, -1.95]
**Subtotal (95% CI)**			**88**			**32**	**68.9%**	**-1.28 [-2.06, -0.50]**
Heterogeneity: Tau² = 0.85; Chi² = 21.93, df = 9 (P = 0.009); I² = 59%								
Test for overall effect: Z = 3.20 (P = 0.001)								
**1.1.2 DHEA**								
BSZY-H 2021	77.91	20.15	10	110.15	30.9	5	9.4%	-1.27 [-2.46, -0.07]
BSZY-L 2021	107.46	28.21	10	110.15	30.9	5	10.2%	-0.09 [-1.16, 0.99]
**Subtotal (95% CI)**			**20**			**10**	**19.6%**	**-0.65 [-1.80, 0.51]**
Heterogeneity: Tau² = 0.36; Chi² = 2.06, df = 1 (P = 0.15); I² = 52%								
Test for overall effect: Z = 1.10 (P = 0.27)								
**1.1.3 Testosterone propionate**								
DXB-2030 2019	57.95	13.41	10	67.49	19	10	11.5%	-0.56 [-1.45, 0.34]
**Subtotal (95% CI)**			**10**			**10**	**11.5%**	**-0.56 [-1.45, 0.34]**
Heterogeneity: Not applicable								
Test for overall effect: Z = 1.21 (P = 0.22)								
**Total (95% CI)**			**118**			**52**	**100.0%**	**-1.01 [-1.58, -0.45]**
Heterogeneity: Tau² = 0.51; Chi² = 25.06, df = 12 (P = 0.01); I² = 52%								
Test for overall effect: Z = 3.52 (P = 0.0004)								
Test for subgroup differences: Chi² = 1.65, df = 2 (P = 0.44), I² = 0%								

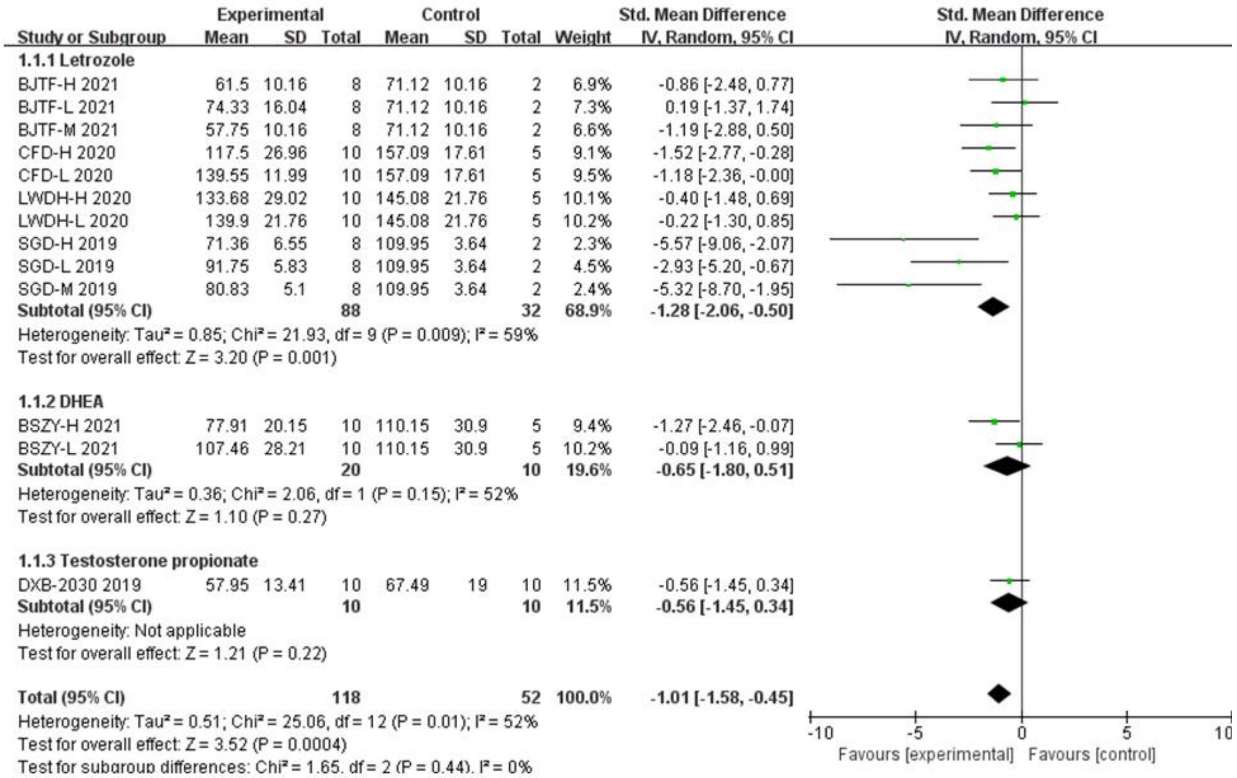

B

Study or Subgroup	Experimental Mean	Experimental SD	Experimental Total	Control Mean	Control SD	Control Total	Weight	Std. Mean Difference IV, Random, 95% CI
**1.2.1 Bushen Jieyu Tiaochong Formula(BJTF)**								
BJTF-H 2021	61.5	10.16	8	71.12	10.16	2	6.9%	-0.86 [-2.48, 0.77]
BJTF-L 2021	74.33	16.04	8	71.12	10.16	2	7.3%	0.19 [-1.37, 1.74]
BJTF-M 2021	57.75	10.16	8	71.12	10.16	2	6.6%	-1.19 [-2.88, 0.50]
**Subtotal (95% CI)**			**24**			**6**	**20.9%**	**-0.58 [-1.51, 0.35]**
Heterogeneity: Tau^2 = 0.00; Chi^2 = 1.55, df = 2 (P = 0.46); I^2 = 0%								
Test for overall effect: Z = 1.22 (P = 0.22)								
**1.2.2 Cangfudaotan Decoction(CFD)**								
CFD-H 2020	117.5	26.96	10	157.09	17.61	5	9.1%	-1.52 [-2.77, -0.28]
CFD-L 2020	139.55	11.99	10	157.09	17.61	5	9.5%	-1.18 [-2.36, -0.00]
**Subtotal (95% CI)**			**20**			**10**	**18.5%**	**-1.34 [-2.20, -0.49]**
Heterogeneity: Tau^2 = 0.00; Chi^2 = 0.15, df = 1 (P = 0.70); I^2 = 0%								
Test for overall effect: Z = 3.07 (P = 0.002)								
**1.2.3 Liuwei Dihuang Pills(LWDH)**								
LWDH-H 2020	133.68	29.02	10	145.08	21.76	5	10.1%	-0.40 [-1.48, 0.69]
LWDH-L 2020	139.9	21.76	10	145.08	21.76	5	10.2%	-0.22 [-1.30, 0.85]
**Subtotal (95% CI)**			**20**			**10**	**20.3%**	**-0.31 [-1.08, 0.45]**
Heterogeneity: Tau^2 = 0.00; Chi^2 = 0.05, df = 1 (P = 0.82); I^2 = 0%								
Test for overall effect: Z = 0.79 (P = 0.43)								
**1.2.4 Shaoyao-Gancao Decoction(SGD)**								
SGD 2021	71.36	6.55	8	109.95	3.64	2	2.3%	-5.57 [-9.06, -2.07]
SGD-H 2019	91.75	5.83	8	109.95	3.64	2	4.5%	-2.93 [-5.20, -0.67]
SGD-L 2019	80.83	5.1	8	109.95	3.64	2	2.4%	-5.32 [-8.70, -1.95]
**Subtotal (95% CI)**			**24**			**6**	**9.1%**	**-4.16 [-5.91, -2.40]**
Heterogeneity: Tau^2 = 0.24; Chi^2 = 2.20, df = 2 (P = 0.33); I^2 = 9%								
Test for overall effect: Z = 4.63 (P < 0.00001)								
**1.2.5 Bu-shen-zhu-yun decoction(BSZY)**								
BSZY-H 2021	77.91	20.15	10	110.15	30.9	5	9.4%	-1.27 [-2.46, -0.07]
BSZY-L 2021	107.46	28.21	10	110.15	30.9	5	10.2%	-0.09 [-1.16, 0.99]
**Subtotal (95% CI)**			**20**			**10**	**19.6%**	**-0.65 [-1.80, 0.51]**
Heterogeneity: Tau^2 = 0.36; Chi^2 = 2.06, df = 1 (P = 0.15); I^2 = 52%								
Test for overall effect: Z = 1.10 (P = 0.27)								
**1.2.6 DXB-2030**								
DXB-2030 2019	57.95	13.41	10	67.49	19	10	11.5%	-0.56 [-1.45, 0.34]
**Subtotal (95% CI)**			**10**			**10**	**11.5%**	**-0.56 [-1.45, 0.34]**
Heterogeneity: Not applicable								
Test for overall effect: Z = 1.21 (P = 0.22)								
**Total (95% CI)**			**118**			**52**	**100.0%**	**-1.01 [-1.58, -0.45]**
Heterogeneity: Tau^2 = 0.51; Chi^2 = 25.06, df = 12 (P = 0.01); I^2 = 52%								
Test for overall effect: Z = 3.52 (P = 0.0004)								
Test for subgroup differences: Chi^2 = 17.61, df = 5 (P = 0.003), I^2 = 71.6%								

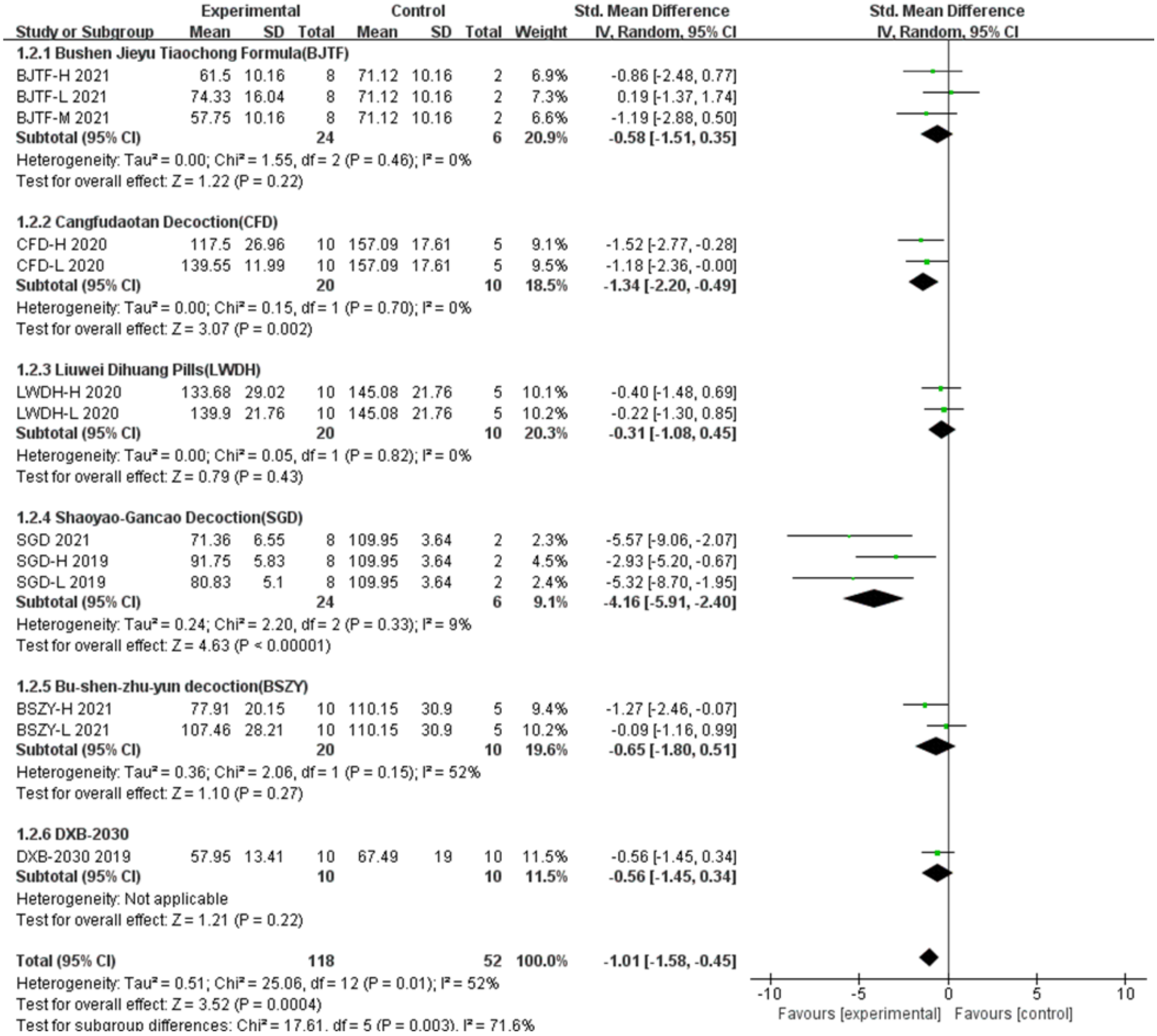

C

Study or Subgroup	Experimental Mean	Experimental SD	Experimental Total	Control Mean	Control SD	Control Total	Weight	Std. Mean Difference IV, Random, 95% CI
**1.3.1 Temperatrure control**								
BJTF-H 2021	61.5	10.16	8	71.12	10.16	2	6.9%	-0.86 [-2.48, 0.77]
BJTF-L 2021	74.33	16.04	8	71.12	10.16	2	7.3%	0.19 [-1.37, 1.74]
BJTF-M 2021	57.75	10.16	8	71.12	10.16	2	6.6%	-1.19 [-2.88, 0.50]
CFD-H 2020	117.5	26.96	10	157.09	17.61	5	9.1%	-1.52 [-2.77, -0.28]
CFD-L 2020	139.55	11.99	10	157.09	17.61	5	9.5%	-1.18 [-2.36, -0.00]
DXB-2030 2019	57.95	13.41	10	67.49	19	10	11.5%	-0.56 [-1.45, 0.34]
LWDH-H 2020	133.68	29.02	10	145.08	21.76	5	10.1%	-0.40 [-1.48, 0.69]
LWDH-L 2020	139.9	21.76	10	145.08	21.76	5	10.2%	-0.22 [-1.30, 0.85]
SGD-H 2019	71.36	6.55	8	109.95	3.64	2	2.3%	-5.57 [-9.06, -2.07]
SGD-L 2019	91.75	5.83	8	109.95	3.64	2	4.5%	-2.93 [-5.20, -0.67]
SGD-M 2019	80.83	5.1	8	109.95	3.64	2	2.4%	-5.32 [-8.70, -1.95]
**Subtotal (95% CI)**			**98**			**42**	**80.4%**	**-1.14 [-1.81, -0.47]**
Heterogeneity: Tau^2 = 0.65; Chi^2 = 22.62, df = 10 (P = 0.01); I^2 = 56%								
Test for overall effect: Z = 3.32 (P = 0.0009)								
**1.3.2 No temperature control**								
BSZY-H 2021	77.91	20.15	10	110.15	30.9	5	9.4%	-1.27 [-2.46, -0.07]
BSZY-L 2021	107.46	28.21	10	110.15	30.9	5	10.2%	-0.09 [-1.16, 0.99]
**Subtotal (95% CI)**			**20**			**10**	**19.6%**	**-0.65 [-1.80, 0.51]**
Heterogeneity: Tau^2 = 0.36; Chi^2 = 2.06, df = 1 (P = 0.15); I^2 = 52%								
Test for overall effect: Z = 1.10 (P = 0.27)								
**Total (95% CI)**			**118**			**52**	**100.0%**	**-1.01 [-1.58, -0.45]**
Heterogeneity: Tau^2 = 0.51; Chi^2 = 25.06, df = 12 (P = 0.01); I^2 = 52%								
Test for overall effect: Z = 3.52 (P = 0.0004)								
Test for subgroup differences: Chi^2 = 0.52, df = 1 (P = 0.47), I^2 = 0%								

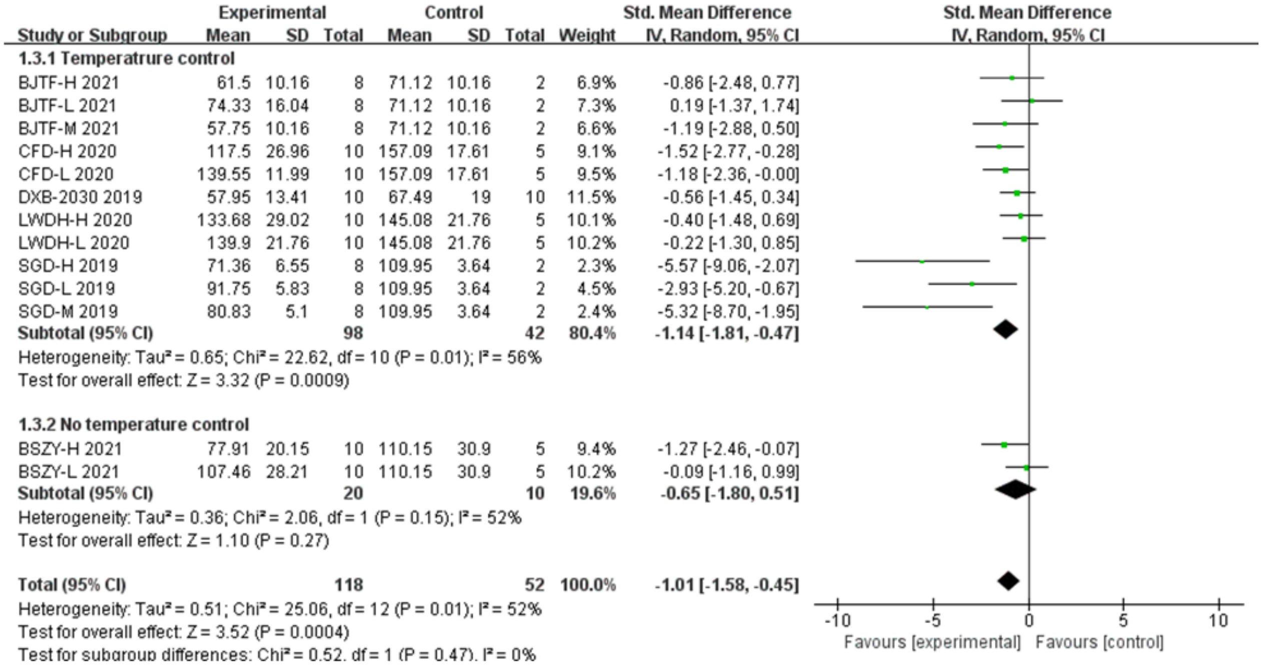

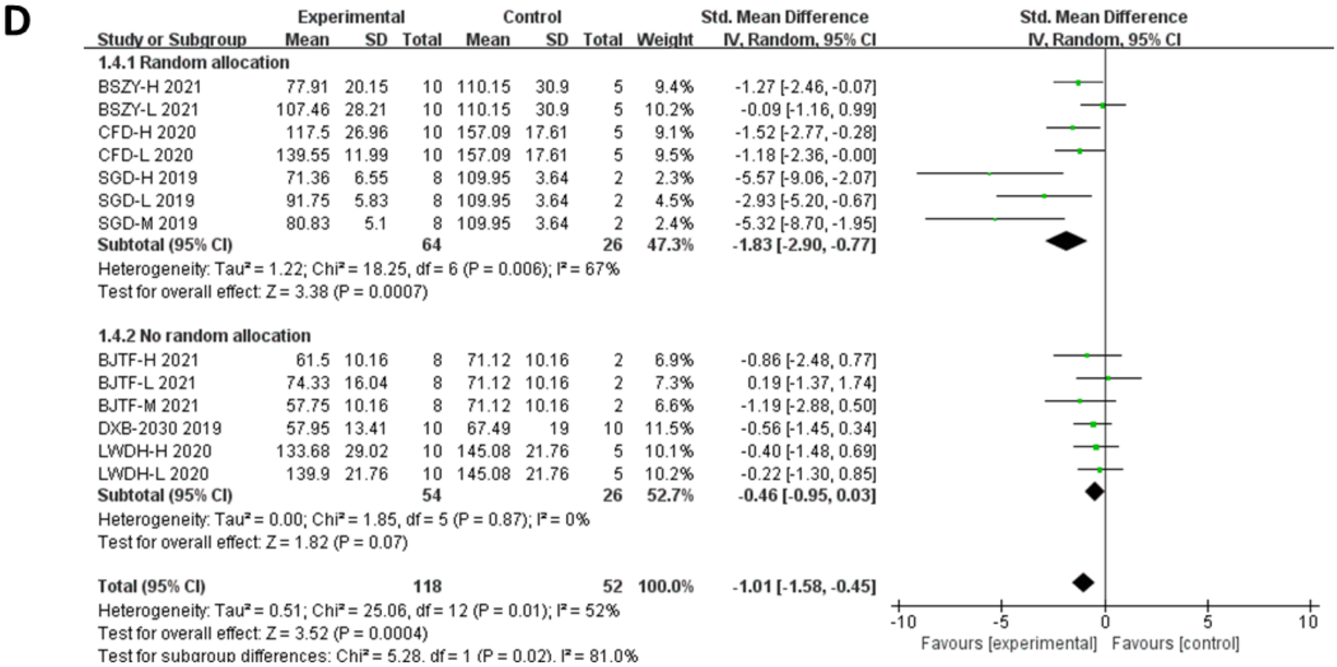

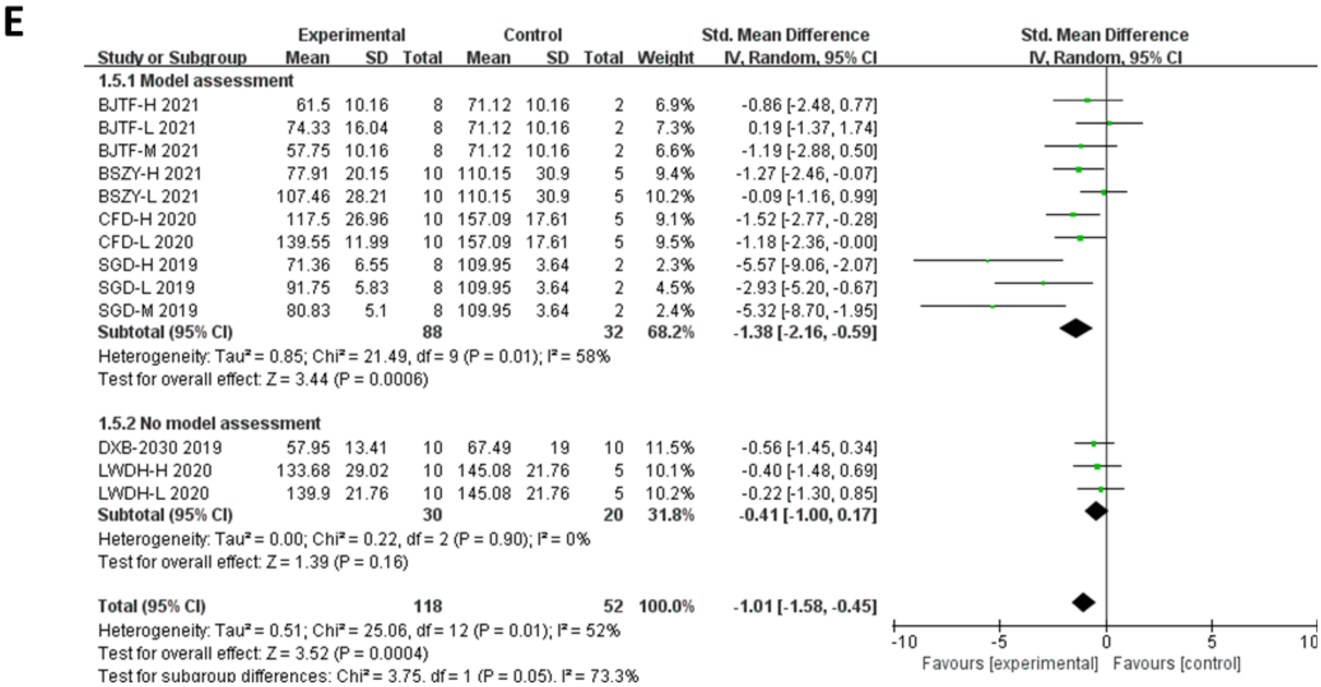

Supplementary Fig. 1 Subgroup analyses were carried out according to (A) PCOS induction drug, (B) formula composition, (C) temperature control, (D) random allocation, and(E) model assessment.

Supplementary Fig. 2 Forest plot of the efficacy of CHM on testosterone

A

Study or Subgroup	Experimental Mean	Experimental SD	Experimental Total	Control Mean	Control SD	Control Total	Weight	Std. Mean Difference IV, Random, 95% CI
**1.6.1 Letrozole**								
BGC-H 2020	12.84	1.4	9	24.87	1.81	4	1.3%	-7.35 [-10.93, -3.77]
BGC-L 2020	14.04	1.2	9	24.87	1.81	4	1.3%	-7.23 [-10.76, -3.70]
BJTF-H 2021	24.44	6.81	8	27.41	8.3	2	3.6%	-0.38 [-1.95, 1.18]
BJTF-L 2021	23.26	7.41	8	27.41	8.3	2	3.6%	-0.50 [-2.07, 1.08]
BJTF-M 2021	19.41	7.7	8	27.41	8.3	2	3.5%	-0.93 [-2.56, 0.71]
CFD-H 2020	60.73	6.31	10	68.61	11.04	5	4.6%	-0.92 [-2.06, 0.22]
CFD-L 2020	59.94	7.89	10	68.61	11.04	5	4.6%	-0.91 [-2.05, 0.23]
FTZ 2021	0.21	0.25	6	0.83	0.3	6	3.7%	-2.07 [-3.59, -0.55]
GFW-H 2020	0.76	0.31	12	1.17	0.28	4	4.3%	-1.28 [-2.52, -0.04]
GFW-H 2021	0.75	0.73	12	1.07	0.9	4	4.5%	-0.39 [-1.54, 0.75]
GFW-L 2020	0.79	0.28	12	1.17	0.28	4	4.3%	-1.28 [-2.53, -0.04]
GFW-L 2021	0.78	0.52	12	1.07	0.9	4	4.5%	-0.44 [-1.59, 0.70]
GFW-M 2020	0.76	0.31	12	1.17	0.28	4	4.3%	-1.28 [-2.52, -0.04]
GFW-M 2021	0.76	0.57	12	1.07	0.9	4	4.5%	-0.45 [-1.59, 0.70]
LWDH-H 2020	1.42	0.31	10	9.66	3.54	5	2.9%	-3.92 [-5.87, -1.96]
LWDH-L 2020	2.38	0.31	10	2.6	0.35	5	4.6%	-0.64 [-1.75, 0.46]
SGD 2021	26.95	8.04	10	34.24	8.84	10	5.1%	-0.83 [-1.75, 0.10]
SGD-H 2019	0.46	0.18	8	0.87	0.25	2	3.0%	-1.95 [-3.85, -0.05]
SGD-L 2019	0.48	0.22	8	0.87	0.25	2	3.2%	-1.57 [-3.36, 0.21]
SGD-M 2019	0.47	0.25	8	0.87	0.25	2	3.3%	-1.45 [-3.20, 0.30]
**Subtotal (95% CI)**			**194**			**80**	**74.8%**	**-1.26 [-1.73, -0.79]**
Heterogeneity: Tau² = 0.57; Chi² = 40.84, df = 19 (P = 0.003); I² = 53%								
Test for overall effect: Z = 5.29 (P < 0.00001)								
**1.6.2 DHEA**								
BHG-H 2021	11.76	11.99	8	56.13	15.59	1	1.8%	-3.29 [-6.19, -0.39]
BHG-L 2021	9.86	10.79	8	56.13	15.59	1	1.6%	-3.81 [-6.95, -0.68]
BHG-M 2021	7.21	10.79	8	56.13	15.59	1	1.5%	-4.03 [-7.27, -0.79]
HEQI 2017	62.19	23.98	10	95.57	71.95	10	5.1%	-0.60 [-1.50, 0.30]
KW-H 2021	9.48	9.59	8	56.13	15.59	1	1.4%	-4.32 [-7.70, -0.94]
KW-L 2021	9.48	10.79	8	56.13	15.59	1	1.6%	-3.84 [-6.99, -0.69]
KW-M 2021	7.58	7.2	8	56.13	15.59	1	1.0%	-5.99 [-10.23, -1.76]
**Subtotal (95% CI)**			**58**			**16**	**13.9%**	**-3.35 [-5.15, -1.55]**
Heterogeneity: Tau² = 3.61; Chi² = 18.69, df = 6 (P = 0.005); I² = 68%								
Test for overall effect: Z = 3.65 (P = 0.0003)								
**1.6.3 Testosterone propionate**								
DXB-2030 2019	0.05	0.06	10	0.24	0.2	10	4.9%	-1.23 [-2.21, -0.26]
**Subtotal (95% CI)**			**10**			**10**	**4.9%**	**-1.23 [-2.21, -0.26]**
Heterogeneity: Not applicable								
Test for overall effect: Z = 2.48 (P = 0.01)								
**1.6.4 Prastenone sodium sulfate**								
SJD-H 2016	1.85	0.23	8	4.98	0.46	2	0.5%	-10.48 [-16.58, -4.38]
SJD-L 2016	3.95	0.32	9	4.98	0.46	2	2.7%	-2.78 [-4.89, -0.67]
SJD-M 2016	4.11	0.42	9	4.98	0.46	2	3.2%	-1.87 [-3.69, -0.06]
**Subtotal (95% CI)**			**26**			**6**	**6.3%**	**-3.63 [-6.59, -0.67]**
Heterogeneity: Tau² = 4.43; Chi² = 7.05, df = 2 (P = 0.03); I² = 72%								
Test for overall effect: Z = 2.41 (P = 0.02)								
**Total (95% CI)**			**288**			**112**	**100.0%**	**-1.62 [-2.07, -1.16]**
Heterogeneity: Tau² = 0.84; Chi² = 73.11, df = 30 (P < 0.0001); I² = 59%								
Test for overall effect: Z = 6.99 (P < 0.00001)								
Test for subgroup differences: Chi² = 7.15, df = 3 (P = 0.07), I² = 58.0%								

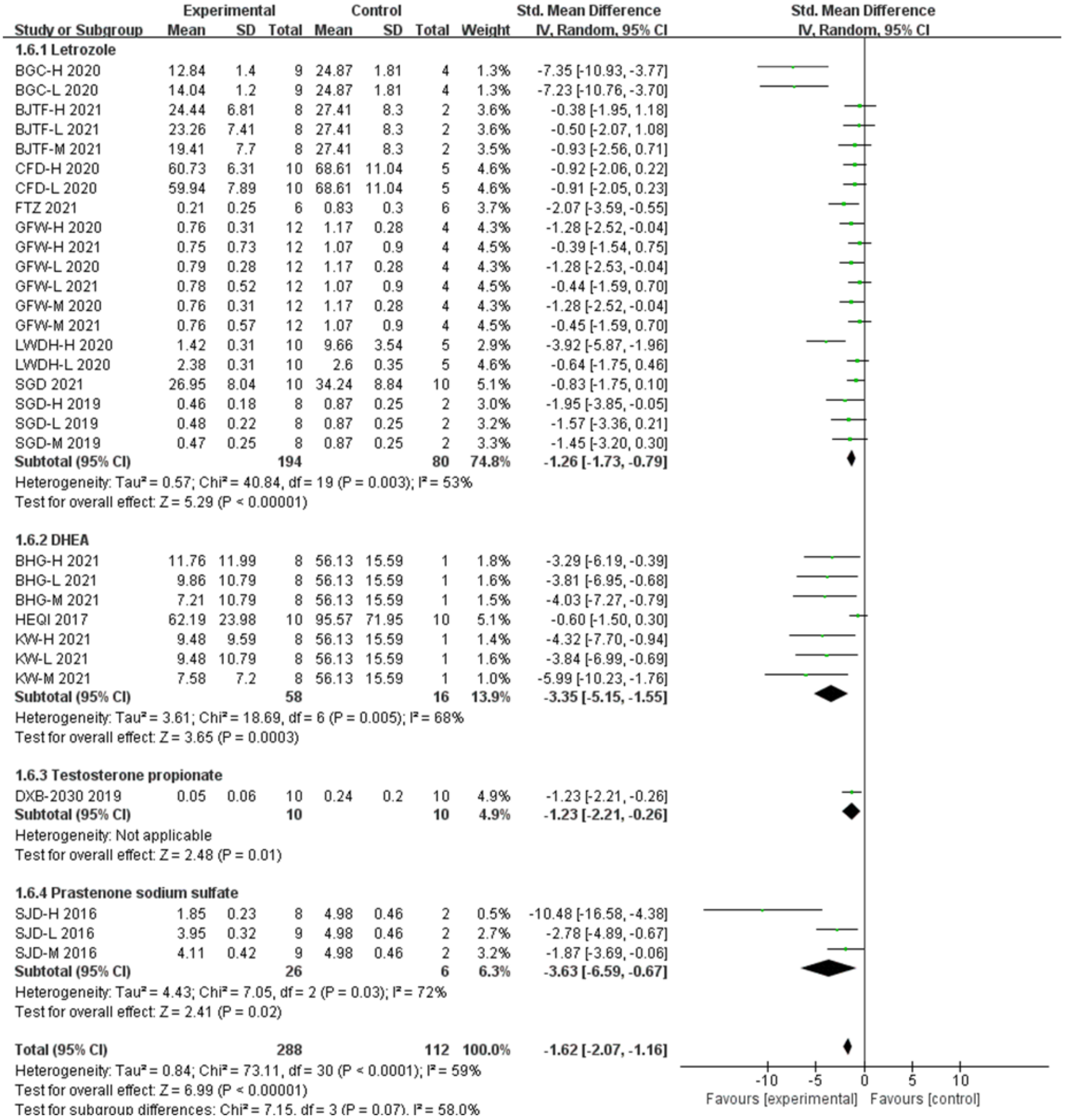

B

Study or Subgroup	Experimental Mean	Experimental SD	Experimental Total	Control Mean	Control SD	Control Total	Weight	Std. Mean Difference IV, Random, 95% CI
**1.7.1 Bushen Jieyu Tiaochong Formula(BJTF)**								
BJTF-H 2021	24.44	6.81	8	27.41	8.3	2	3.6%	-0.38 [-1.95, 1.18]
BJTF-L 2021	23.26	7.41	8	27.41	8.3	2	3.6%	-0.50 [-2.07, 1.08]
BJTF-M 2021	19.41	7.7	8	27.41	8.3	2	3.5%	-0.93 [-2.56, 0.71]
**Subtotal (95% CI)**			**24**			**6**	**10.7%**	**-0.59 [-1.51, 0.32]**
Heterogeneity: Tau² = 0.00; Chi² = 0.25, df = 2 (P = 0.88); I² = 0%								
Test for overall effect: Z = 1.27 (P = 0.20)								
**1.7.2 Shaoyao-Gancao Decoction(SGD)**								
SGD 2021	26.95	8.04	10	34.24	8.84	10	5.1%	-0.83 [-1.75, 0.10]
SGD-H 2019	0.46	0.18	8	0.87	0.25	2	3.0%	-1.95 [-3.85, -0.05]
SGD-L 2019	0.48	0.22	8	0.87	0.25	2	3.2%	-1.57 [-3.36, 0.21]
SGD-M 2019	0.47	0.25	8	0.87	0.25	2	3.3%	-1.45 [-3.20, 0.30]
**Subtotal (95% CI)**			**34**			**16**	**14.6%**	**-1.18 [-1.87, -0.49]**
Heterogeneity: Tau² = 0.00; Chi² = 1.47, df = 3 (P = 0.69); I² = 0%								
Test for overall effect: Z = 3.36 (P = 0.0008)								
**1.7.3 Bushen Huatan Granules(BHG)**								
BHG-H 2021	11.76	11.99	8	56.13	15.59	1	1.8%	-3.29 [-6.19, -0.39]
BHG-L 2021	9.86	10.79	8	56.13	15.59	1	1.6%	-3.81 [-6.95, -0.68]
BHG-M 2021	7.21	10.79	8	56.13	15.59	1	1.5%	-4.03 [-7.27, -0.79]
**Subtotal (95% CI)**			**24**			**3**	**4.8%**	**-3.68 [-5.46, -1.90]**
Heterogeneity: Tau² = 0.00; Chi² = 0.12, df = 2 (P = 0.94); I² = 0%								
Test for overall effect: Z = 4.05 (P < 0.0001)								
**1.7.4 Kunling Wan(KW)**								
KW-H 2021	9.48	9.59	8	56.13	15.59	1	1.4%	-4.32 [-7.70, -0.94]
KW-L 2021	9.48	10.79	8	56.13	15.59	1	1.6%	-3.84 [-6.99, -0.69]
KW-M 2021	7.58	7.2	8	56.13	15.59	1	1.0%	-5.99 [-10.23, -1.76]
**Subtotal (95% CI)**			**24**			**3**	**3.9%**	**-4.51 [-6.53, -2.48]**
Heterogeneity: Tau² = 0.00; Chi² = 0.65, df = 2 (P = 0.72); I² = 0%								
Test for overall effect: Z = 4.36 (P < 0.0001)								
**1.7.5 Guizhi Fuling Wan(GFW)**								
GFW-H 2020	0.76	0.31	12	1.17	0.28	4	4.3%	-1.28 [-2.52, -0.04]
GFW-H 2021	0.75	0.73	12	1.07	0.9	4	4.5%	-0.39 [-1.54, 0.75]
GFW-L 2020	0.79	0.28	12	1.17	0.28	4	4.3%	-1.28 [-2.53, -0.04]
GFW-L 2021	0.78	0.52	12	1.07	0.9	4	4.5%	-0.44 [-1.59, 0.70]
GFW-M 2020	0.76	0.31	12	1.17	0.28	4	4.3%	-1.28 [-2.52, -0.04]
GFW-M 2021	0.76	0.57	12	1.07	0.9	4	4.5%	-0.45 [-1.59, 0.70]
**Subtotal (95% CI)**			**72**			**24**	**26.6%**	**-0.82 [-1.30, -0.33]**
Heterogeneity: Tau² = 0.00; Chi² = 2.93, df = 5 (P = 0.71); I² = 0%								
Test for overall effect: Z = 3.30 (P = 0.0010)								
**1.7.6 Cangfudaotan Decoction(CFD)**								
CFD-H 2020	60.73	6.31	10	68.61	11.04	5	4.6%	-0.92 [-2.06, 0.22]
CFD-L 2020	59.94	7.89	10	68.61	11.04	5	4.6%	-0.91 [-2.05, 0.23]
**Subtotal (95% CI)**			**20**			**10**	**9.1%**	**-0.91 [-1.72, -0.11]**
Heterogeneity: Tau² = 0.00; Chi² = 0.00, df = 1 (P = 0.99); I² = 0%								
Test for overall effect: Z = 2.22 (P = 0.03)								
**1.7.7 FuFang ZhenZhu TiaoZi(FTZ)**								
FTZ 2021	0.21	0.25	6	0.83	0.3	6	3.7%	-2.07 [-3.59, -0.55]
**Subtotal (95% CI)**			**6**			**6**	**3.7%**	**-2.07 [-3.59, -0.55]**
Heterogeneity: Not applicable								
Test for overall effect: Z = 2.68 (P = 0.007)								
**1.7.8 Bao Gui capsule(BGC)**								
BGC-H 2020	12.84	1.4	9	24.87	1.81	4	1.3%	-7.35 [-10.93, -3.77]
BGC-L 2020	14.04	1.2	9	24.87	1.81	4	1.3%	-7.23 [-10.76, -3.70]
**Subtotal (95% CI)**			**18**			**8**	**2.6%**	**-7.29 [-9.80, -4.77]**
Heterogeneity: Tau² = 0.00; Chi² = 0.00, df = 1 (P = 0.96); I² = 0%								
Test for overall effect: Z = 5.68 (P < 0.00001)								
**1.7.9 Liuwei Dihuang Pills(LWDH)**								
LWDH-H 2020	1.42	0.31	10	9.66	3.54	5	2.9%	-3.92 [-5.87, -1.96]
LWDH-L 2020	2.38	0.31	10	2.6	0.35	5	4.6%	-0.64 [-1.75, 0.46]
**Subtotal (95% CI)**			**20**			**10**	**7.6%**	**-2.18 [-5.38, 1.03]**
Heterogeneity: Tau² = 4.71; Chi² = 8.17, df = 1 (P = 0.004); I² = 88%								
Test for overall effect: Z = 1.33 (P = 0.18)								
**1.7.10 DXB-2030**								
DXB-2030 2019	0.05	0.06	10	0.24	0.2	10	4.9%	-1.23 [-2.21, -0.26]
**Subtotal (95% CI)**			**10**			**10**	**4.9%**	**-1.23 [-2.21, -0.26]**
Heterogeneity: Not applicable								
Test for overall effect: Z = 2.48 (P = 0.01)								
**1.7.11 Heqi San(HEQI)**								
HEQI 2017	62.19	23.98	10	95.57	71.95	10	5.1%	-0.60 [-1.50, 0.30]
**Subtotal (95% CI)**			**10**			**10**	**5.1%**	**-0.60 [-1.50, 0.30]**
Heterogeneity: Not applicable								
Test for overall effect: Z = 1.30 (P = 0.19)								
**1.7.12 Shouwu Jiangqi Decoction(SJD)**								
SJD-H 2016	1.85	0.23	8	4.98	0.46	2	0.5%	-10.48 [-16.58, -4.38]
SJD-L 2016	3.95	0.32	9	4.98	0.46	2	2.7%	-2.78 [-4.89, -0.67]
SJD-M 2016	4.11	0.42	9	4.98	0.46	2	3.2%	-1.87 [-3.69, -0.06]
**Subtotal (95% CI)**			**26**			**6**	**6.3%**	**-3.63 [-6.59, -0.67]**
Heterogeneity: Tau² = 4.43; Chi² = 7.05, df = 2 (P = 0.03); I² = 72%								
Test for overall effect: Z = 2.41 (P = 0.02)								
**Total (95% CI)**			**288**			**112**	**100.0%**	**-1.62 [-2.07, -1.16]**
Heterogeneity: Tau² = 0.84; Chi² = 73.11, df = 30 (P < 0.0001); I² = 59%								
Test for overall effect: Z = 6.99 (P < 0.00001)								
Test for subgroup differences: Chi² = 50.70, df = 11 (P < 0.00001), I² = 78.3%								

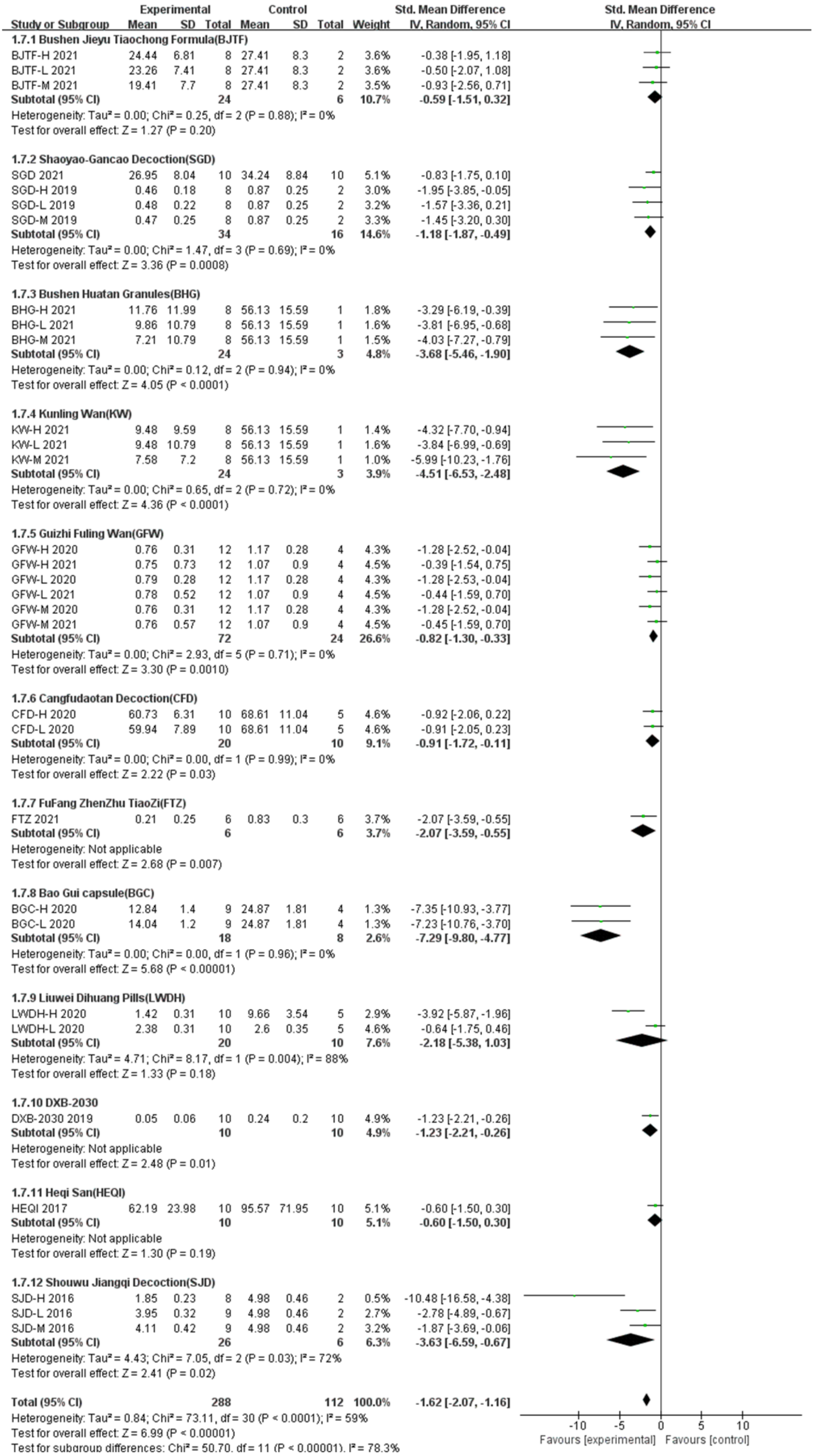

C

Study or Subgroup	Experimental Mean	Experimental SD	Experimental Total	Control Mean	Control SD	Control Total	Weight	Std. Mean Difference IV, Random, 95% CI
**1.8.1 Temperatrure control**								
BGC-H 2020	12.84	1.4	9	24.87	1.81	4	1.3%	-7.35 [-10.93, -3.77]
BGC-L 2020	14.04	1.2	9	24.87	1.81	4	1.3%	-7.23 [-10.76, -3.70]
BHG-H 2021	11.76	11.99	8	56.13	15.59	1	1.8%	-3.29 [-6.19, -0.39]
BHG-L 2021	9.86	10.79	8	56.13	15.59	1	1.6%	-3.81 [-6.95, -0.68]
BHG-M 2021	7.21	10.79	8	56.13	15.59	1	1.5%	-4.03 [-7.27, -0.79]
BJTF-H 2021	24.44	6.81	8	27.41	8.3	2	3.6%	-0.38 [-1.95, 1.18]
BJTF-L 2021	23.26	7.41	8	27.41	8.3	2	3.6%	-0.50 [-2.07, 1.08]
BJTF-M 2021	19.41	7.7	8	27.41	8.3	2	3.5%	-0.93 [-2.56, 0.71]
CFD-H 2020	60.73	6.31	10	68.61	11.04	5	4.6%	-0.92 [-2.06, 0.22]
CFD-L 2020	59.94	7.89	10	68.61	11.04	5	4.6%	-0.91 [-2.05, 0.23]
DXB-2030 2019	0.05	0.06	10	0.24	0.2	10	4.9%	-1.23 [-2.21, -0.26]
FTZ 2021	0.21	0.25	6	0.83	0.3	6	3.7%	-2.07 [-3.59, -0.55]
GFW-H 2021	0.75	0.73	12	1.07	0.9	4	4.5%	-0.39 [-1.54, 0.75]
GFW-L 2021	0.78	0.52	12	1.07	0.9	4	4.5%	-0.44 [-1.59, 0.70]
GFW-M 2021	0.76	0.57	12	1.07	0.9	4	4.5%	-0.45 [-1.59, 0.70]
HEQI 2017	62.19	23.98	10	95.57	71.95	10	5.1%	-0.60 [-1.50, 0.30]
KW-H 2021	9.48	9.59	8	56.13	15.59	1	1.4%	-4.32 [-7.70, -0.94]
KW-L 2021	9.48	10.79	8	56.13	15.59	1	1.6%	-3.84 [-6.99, -0.69]
KW-M 2021	7.58	7.2	8	56.13	15.59	1	1.0%	-5.99 [-10.23, -1.76]
LWDH-H 2020	1.42	0.31	10	9.66	3.54	5	2.9%	-3.92 [-5.87, -1.96]
LWDH-L 2020	2.38	0.31	10	2.6	0.35	5	4.6%	-0.64 [-1.75, 0.46]
SGD 2021	26.95	8.04	10	34.24	8.84	10	5.1%	-0.83 [-1.75, 0.10]
SGD-H 2019	0.46	0.18	8	0.87	0.25	2	3.0%	-1.95 [-3.85, -0.05]
SGD-L 2019	0.48	0.22	8	0.87	0.25	2	3.2%	-1.57 [-3.36, 0.21]
SGD-M 2019	0.47	0.25	8	0.87	0.25	2	3.3%	-1.45 [-3.20, 0.30]
**Subtotal (95% CI)**			**226**			**94**	**80.7%**	**-1.58 [-2.10, -1.07]**
Heterogeneity: Tau² = 0.90; Chi² = 61.47, df = 24 (P < 0.0001); I² = 61%								
Test for overall effect: Z = 6.02 (P < 0.00001)								
**1.8.2 No temperature control**								
GFW-H 2020	0.76	0.31	12	1.17	0.28	4	4.3%	-1.28 [-2.52, -0.04]
GFW-L 2020	0.79	0.28	12	1.17	0.28	4	4.3%	-1.28 [-2.53, -0.04]
GFW-M 2020	0.76	0.31	12	1.17	0.28	4	4.3%	-1.28 [-2.52, -0.04]
SJD-H 2016	1.85	0.23	8	4.98	0.46	2	0.5%	-10.48 [-16.58, -4.38]
SJD-L 2016	3.95	0.32	9	4.98	0.46	2	2.7%	-2.78 [-4.89, -0.67]
SJD-M 2016	4.11	0.42	9	4.98	0.46	2	3.2%	-1.87 [-3.69, -0.06]
**Subtotal (95% CI)**			**62**			**18**	**19.3%**	**-1.80 [-2.78, -0.82]**
Heterogeneity: Tau² = 0.70; Chi² = 10.21, df = 5 (P = 0.07); I² = 51%								
Test for overall effect: Z = 3.60 (P = 0.0003)								
**Total (95% CI)**			**288**			**112**	**100.0%**	**-1.62 [-2.07, -1.16]**
Heterogeneity: Tau² = 0.84; Chi² = 73.11, df = 30 (P < 0.0001); I² = 59%								
Test for overall effect: Z = 6.99 (P < 0.00001)								
Test for subgroup differences: Chi² = 0.15, df = 1 (P = 0.70), I² = 0%								

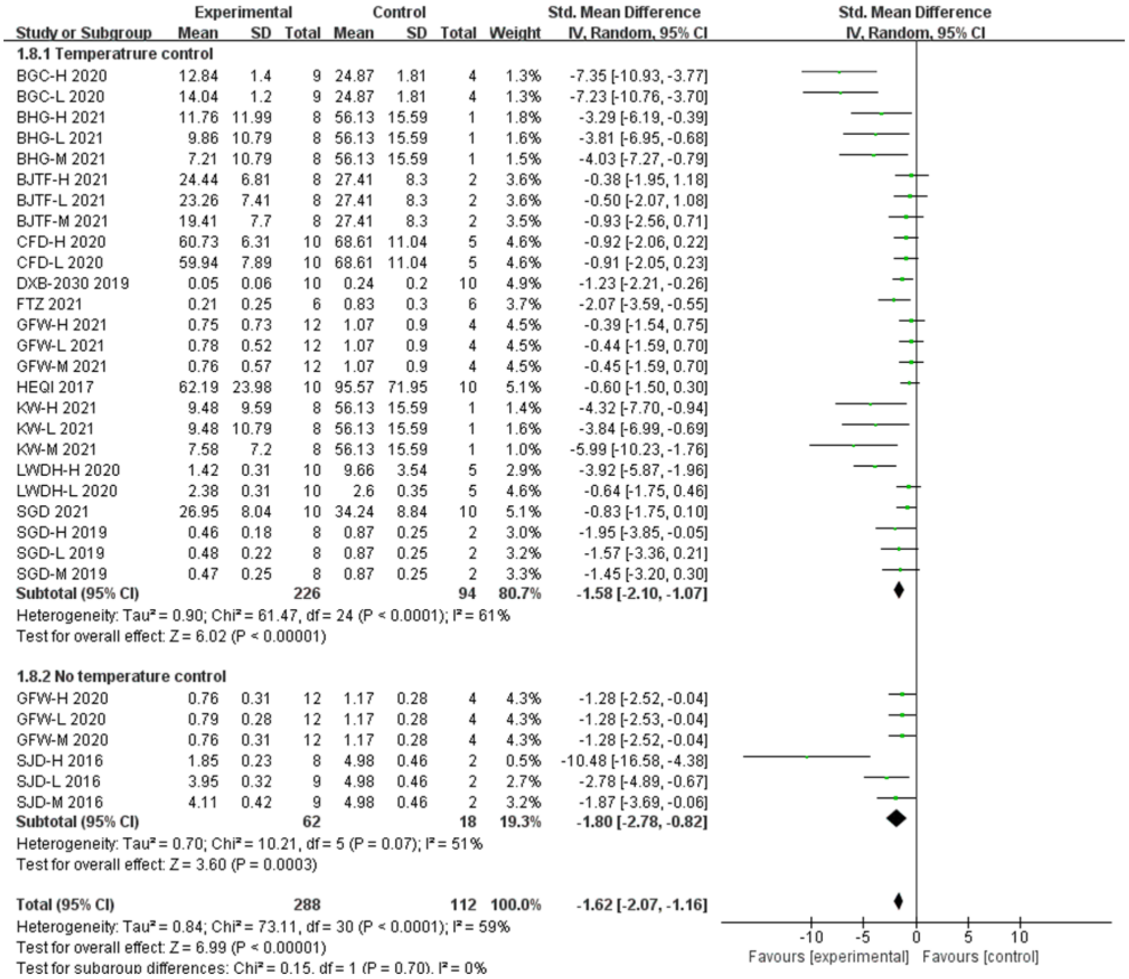

D

Study or Subgroup	Experimental Mean	Experimental SD	Experimental Total	Control Mean	Control SD	Control Total	Weight	Std. Mean Difference IV, Random, 95% CI
**1.9.1 Random allocation**								
BGC-H 2020	12.84	1.4	9	24.87	1.81	4	1.3%	-7.35 [-10.93, -3.77]
BGC-L 2020	14.04	1.2	9	24.87	1.81	4	1.3%	-7.23 [-10.76, -3.70]
CFD-H 2020	60.73	6.31	10	68.61	11.04	5	4.6%	-0.92 [-2.06, 0.22]
CFD-L 2020	59.94	7.89	10	68.61	11.04	5	4.6%	-0.91 [-2.05, 0.23]
GFW-H 2020	0.76	0.31	12	1.17	0.28	4	4.3%	-1.28 [-2.52, -0.04]
GFW-H 2021	0.75	0.73	12	1.07	0.9	4	4.5%	-0.39 [-1.54, 0.75]
GFW-L 2020	0.79	0.28	12	1.17	0.28	4	4.3%	-1.28 [-2.53, -0.04]
GFW-L 2021	0.78	0.52	12	1.07	0.9	4	4.5%	-0.44 [-1.59, 0.70]
GFW-M 2020	0.76	0.31	12	1.17	0.28	4	4.3%	-1.28 [-2.52, -0.04]
GFW-M 2021	0.76	0.57	12	1.07	0.9	4	4.5%	-0.45 [-1.59, 0.70]
HEQI 2017	62.19	23.98	10	95.57	71.95	10	5.1%	-0.60 [-1.50, 0.30]
SGD 2021	26.95	8.04	10	34.24	8.84	10	5.1%	-0.83 [-1.75, 0.10]
SGD-H 2019	0.46	0.18	8	0.87	0.25	2	3.0%	-1.95 [-3.85, -0.05]
SGD-L 2019	0.48	0.22	8	0.87	0.25	2	3.2%	-1.57 [-3.36, 0.21]
SGD-M 2019	0.47	0.25	8	0.87	0.25	2	3.3%	-1.45 [-3.20, 0.30]
SJD-H 2016	1.85	0.23	8	4.98	0.46	2	0.5%	-10.48 [-16.58, -4.38]
SJD-L 2016	3.95	0.32	9	4.98	0.46	2	2.7%	-2.78 [-4.89, -0.67]
SJD-M 2016	4.11	0.42	9	4.98	0.46	2	3.2%	-1.87 [-3.69, -0.06]
**Subtotal (95% CI)**			**180**			**74**	**64.3%**	**-1.39 [-1.94, -0.85]**
Heterogeneity: Tau² = 0.74; Chi² = 42.64, df = 17 (P = 0.0005); I² = 60%								
Test for overall effect: Z = 5.02 (P < 0.00001)								
**1.9.2 No random allocation**								
BHG-H 2021	11.76	11.99	8	56.13	15.59	1	1.8%	-3.29 [-6.19, -0.39]
BHG-L 2021	9.86	10.79	8	56.13	15.59	1	1.6%	-3.81 [-6.95, -0.68]
BHG-M 2021	7.21	10.79	8	56.13	15.59	1	1.5%	-4.03 [-7.27, -0.79]
BJTF-H 2021	24.44	6.81	8	27.41	8.3	2	3.6%	-0.38 [-1.95, 1.18]
BJTF-L 2021	23.26	7.41	8	27.41	8.3	2	3.6%	-0.50 [-2.07, 1.08]
BJTF-M 2021	19.41	7.7	8	27.41	8.3	2	3.5%	-0.93 [-2.56, 0.71]
DXB-2030 2019	0.05	0.06	10	0.24	0.2	10	4.9%	-1.23 [-2.21, -0.26]
FTZ 2021	0.21	0.25	6	0.83	0.3	6	3.7%	-2.07 [-3.59, -0.55]
KW-H 2021	9.48	9.59	8	56.13	15.59	1	1.4%	-4.32 [-7.70, -0.94]
KW-L 2021	9.48	10.79	8	56.13	15.59	1	1.6%	-3.84 [-6.99, -0.69]
KW-M 2021	7.58	7.2	8	56.13	15.59	1	1.0%	-5.99 [-10.23, -1.76]
LWDH-H 2020	1.42	0.31	10	9.66	3.54	5	2.9%	-3.92 [-5.87, -1.96]
LWDH-L 2020	2.38	0.31	10	2.6	0.35	5	4.6%	-0.64 [-1.75, 0.46]
**Subtotal (95% CI)**			**108**			**38**	**35.7%**	**-2.04 [-2.86, -1.22]**
Heterogeneity: Tau² = 1.12; Chi² = 27.97, df = 12 (P = 0.006); I² = 57%								
Test for overall effect: Z = 4.87 (P < 0.00001)								
**Total (95% CI)**			**288**			**112**	**100.0%**	**-1.62 [-2.07, -1.16]**
Heterogeneity: Tau² = 0.84; Chi² = 73.11, df = 30 (P < 0.0001); I² = 59%								
Test for overall effect: Z = 6.99 (P < 0.00001)								
Test for subgroup differences: Chi² = 1.67, df = 1 (P = 0.20), I² = 40.2%								

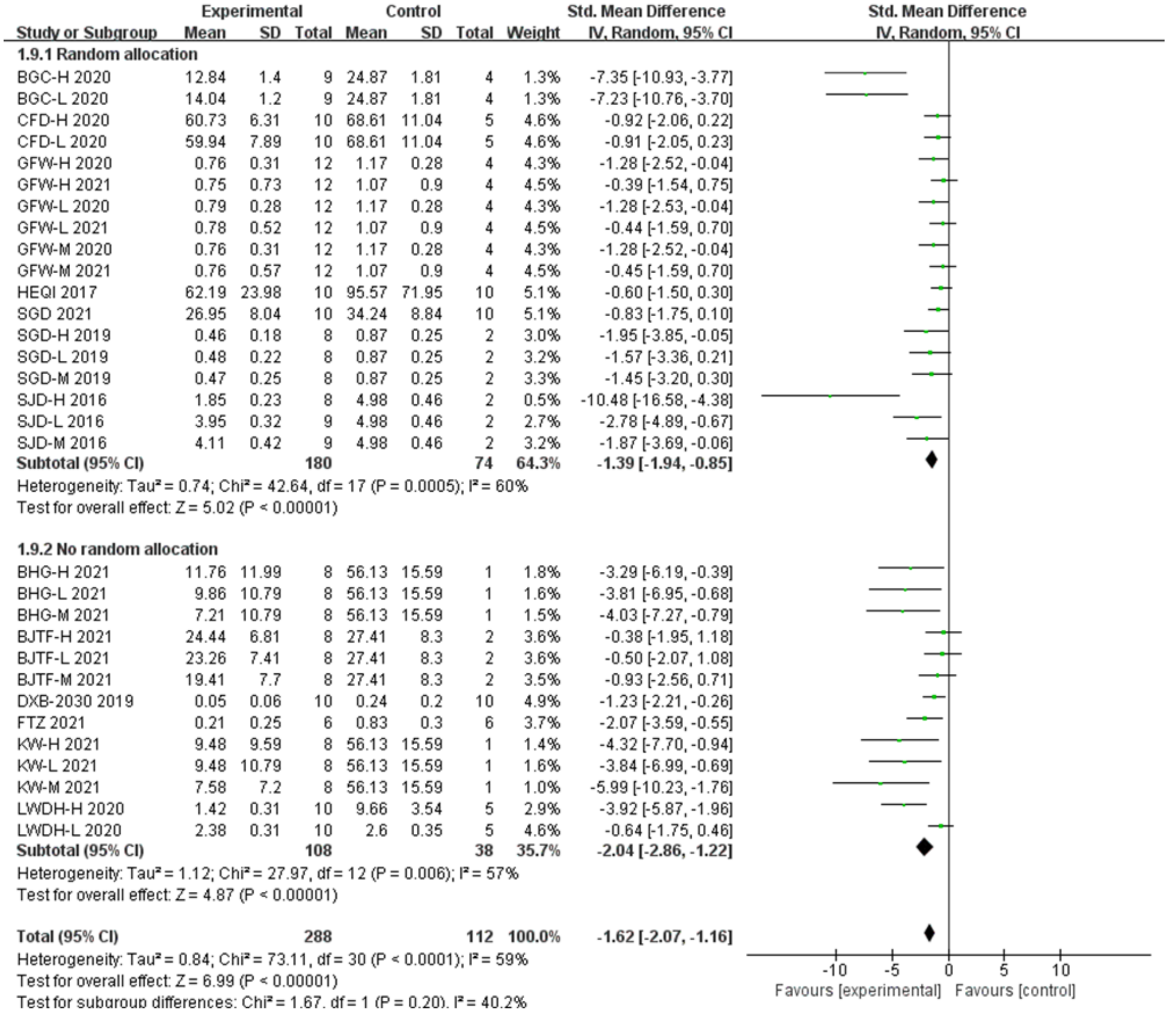

E

	Experimental			Control				Std. Mean Difference
Study or Subgroup	Mean	SD	Total	Mean	SD	Total	Weight	IV, Random, 95% CI
**1.10.1 Model assessment**								
BGC-H 2020	12.84	1.4	9	24.87	1.81	4	1.3%	-7.35 [-10.93, -3.77]
BGC-L 2020	14.04	1.2	9	24.87	1.81	4	1.3%	-7.23 [-10.76, -3.70]
BJTF-H 2021	24.44	6.81	8	27.41	8.3	2	3.6%	-0.38 [-1.95, 1.18]
BJTF-L 2021	23.26	7.41	8	27.41	8.3	2	3.6%	-0.50 [-2.07, 1.08]
BJTF-M 2021	19.41	7.7	8	27.41	8.3	2	3.5%	-0.93 [-2.56, 0.71]
CFD-H 2020	60.73	6.31	10	68.61	11.04	5	4.6%	-0.92 [-2.06, 0.22]
CFD-L 2020	59.94	7.89	10	68.61	11.04	5	4.6%	-0.91 [-2.05, 0.23]
FTZ 2021	0.21	0.25	6	0.83	0.3	6	3.7%	-2.07 [-3.59, -0.55]
SGD-H 2019	0.46	0.18	8	0.87	0.25	2	3.0%	-1.95 [-3.85, -0.05]
SGD-L 2019	0.48	0.22	8	0.87	0.25	2	3.2%	-1.57 [-3.36, 0.21]
SGD-M 2019	0.47	0.25	8	0.87	0.25	2	3.3%	-1.45 [-3.20, 0.30]
SJD-H 2016	1.85	0.23	8	4.98	0.46	2	0.5%	-10.48 [-16.58, -4.38]
SJD-L 2016	3.95	0.32	9	4.98	0.46	2	2.7%	-2.78 [-4.89, -0.67]
SJD-M 2016	4.11	0.42	9	4.98	0.46	2	3.2%	-1.87 [-3.69, -0.06]
**Subtotal (95% CI)**			**118**			**42**	**42.0%**	**-1.93 [-2.76, -1.11]**
Heterogeneity: Tau² = 1.45; Chi² = 37.05, df = 13 (P = 0.0004); I² = 65%								
Test for overall effect: Z = 4.59 (P < 0.00001)								
**1.10.2 No model assessment**								
BHG-H 2021	11.76	11.99	8	56.13	15.59	1	1.8%	-3.29 [-6.19, -0.39]
BHG-L 2021	9.86	10.79	8	56.13	15.59	1	1.6%	-3.81 [-6.95, -0.68]
BHG-M 2021	7.21	10.79	8	56.13	15.59	1	1.5%	-4.03 [-7.27, -0.79]
DXB-2030 2019	0.05	0.06	10	0.24	0.2	10	4.9%	-1.23 [-2.21, -0.26]
GFW-H 2020	0.76	0.31	12	1.17	0.28	4	4.3%	-1.28 [-2.52, -0.04]
GFW-H 2021	0.75	0.73	12	1.07	0.9	4	4.5%	-0.39 [-1.54, 0.75]
GFW-L 2020	0.79	0.28	12	1.17	0.28	4	4.3%	-1.28 [-2.53, -0.04]
GFW-L 2021	0.78	0.52	12	1.07	0.9	4	4.5%	-0.44 [-1.59, 0.70]
GFW-M 2020	0.76	0.31	12	1.17	0.28	4	4.3%	-1.28 [-2.52, -0.04]
GFW-M 2021	0.76	0.57	12	1.07	0.9	4	4.5%	-0.45 [-1.59, 0.70]
HEQI 2017	62.19	23.98	10	95.57	71.95	10	5.1%	-0.60 [-1.50, 0.30]
KW-H 2021	9.48	9.59	8	56.13	15.59	1	1.4%	-4.32 [-7.70, -0.94]
KW-L 2021	9.48	10.79	8	56.13	15.59	1	1.6%	-3.84 [-6.99, -0.69]
KW-M 2021	7.58	7.2	8	56.13	15.59	1	1.0%	-5.99 [-10.23, -1.76]
LWDH-H 2020	1.42	0.31	10	9.66	3.54	5	2.9%	-3.92 [-5.87, -1.96]
LWDH-L 2020	2.38	0.31	10	2.6	0.35	5	4.6%	-0.64 [-1.75, 0.46]
SGD 2021	26.95	8.04	10	34.24	8.84	10	5.1%	-0.83 [-1.75, 0.10]
**Subtotal (95% CI)**			**170**			**70**	**58.0%**	**-1.39 [-1.92, -0.87]**
Heterogeneity: Tau² = 0.56; Chi² = 34.21, df = 16 (P = 0.005); I² = 53%								
Test for overall effect: Z = 5.18 (P < 0.00001)								
**Total (95% CI)**			**288**			**112**	**100.0%**	**-1.62 [-2.07, -1.16]**
Heterogeneity: Tau² = 0.84; Chi² = 73.11, df = 30 (P < 0.0001); I² = 59%								
Test for overall effect: Z = 6.99 (P < 0.00001)								
Test for subgroup differences: Chi² = 1.17, df = 1 (P = 0.28), I² = 14.6%								

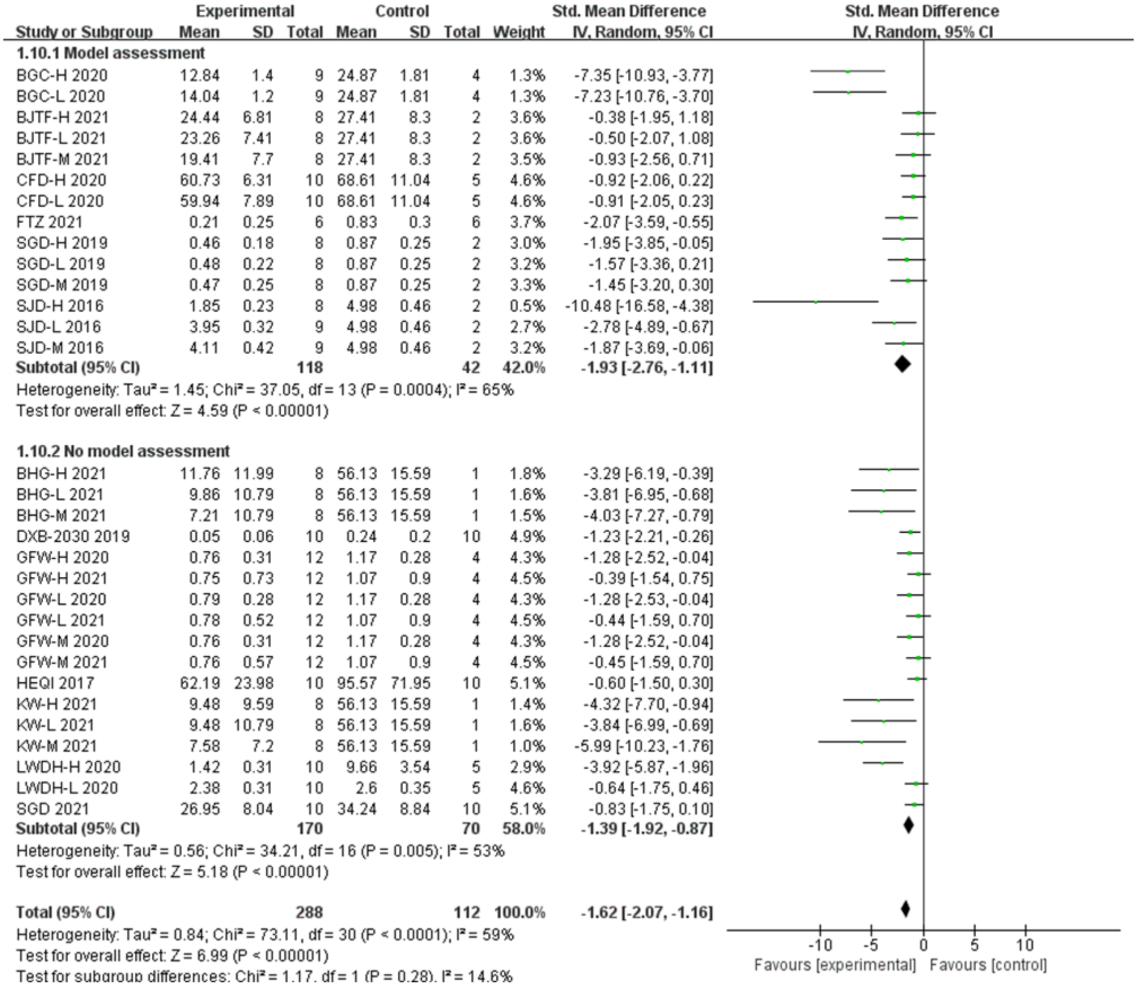

Supplementary Fig. 2 Subgroup analyses were carried out according to (A) PCOS induction drug, (B) formula composition, (C) temperature control, (D) random allocation, and(E) model assessment.

Supplementary Fig. 3 Forest plots of the efficacy of different formulas.

A

Study or Subgroup	Experimental Mean	Experimental SD	Experimental Total	Control Mean	Control SD	Control Total	Weight	Std. Mean Difference IV, Random, 95% CI
**1.7.1 Bushen Jieyu Tiaochong Formula(BJTF)**								
BJTF-H 2021	61.5	10.16	8	71.12	10.16	2	6.9%	-0.86 [-2.48, 0.77]
BJTF-L 2021	74.33	16.04	8	71.12	10.16	2	7.3%	0.19 [-1.37, 1.74]
BJTF-M 2021	57.75	10.16	8	71.12	10.16	2	6.6%	-1.19 [-2.88, 0.50]
**Subtotal (95% CI)**			**24**			**6**	**20.9%**	**-0.58 [-1.51, 0.35]**
Heterogeneity: Tau² = 0.00; Chi² = 1.55, df = 2 (P = 0.46); I² = 0%								
Test for overall effect: Z = 1.22 (P = 0.22)								
**1.7.2 Cangfudaotan Decoction(CFD)**								
CFD-H 2020	117.5	26.96	10	157.09	17.61	5	9.1%	-1.52 [-2.77, -0.28]
CFD-L 2020	139.55	11.99	10	157.09	17.61	5	9.5%	-1.18 [-2.36, -0.00]
**Subtotal (95% CI)**			**20**			**10**	**18.5%**	**-1.34 [-2.20, -0.49]**
Heterogeneity: Tau² = 0.00; Chi² = 0.15, df = 1 (P = 0.70); I² = 0%								
Test for overall effect: Z = 3.07 (P = 0.002)								
**1.7.3 Liuwei Dihuang Pills(LWDH)**								
LWDH-H 2020	133.68	29.02	10	145.08	21.76	5	10.1%	-0.40 [-1.48, 0.69]
LWDH-L 2020	139.9	21.76	10	145.08	21.76	5	10.2%	-0.22 [-1.30, 0.85]
**Subtotal (95% CI)**			**20**			**10**	**20.3%**	**-0.31 [-1.08, 0.45]**
Heterogeneity: Tau² = 0.00; Chi² = 0.05, df = 1 (P = 0.82); I² = 0%								
Test for overall effect: Z = 0.79 (P = 0.43)								
**1.7.4 Shaoyao-Gancao Decoction(SGD)**								
SGD 2021	71.36	6.55	8	109.95	3.64	2	2.3%	-5.57 [-9.06, -2.07]
SGD-H 2019	91.75	5.83	8	109.95	3.64	2	4.5%	-2.93 [-5.20, -0.67]
SGD-L 2019	80.83	5.1	8	109.95	3.64	2	2.4%	-5.32 [-8.70, -1.95]
**Subtotal (95% CI)**			**24**			**6**	**9.1%**	**-4.16 [-5.91, -2.40]**
Heterogeneity: Tau² = 0.24; Chi² = 2.20, df = 2 (P = 0.33); I² = 9%								
Test for overall effect: Z = 4.63 (P < 0.00001)								
**1.7.5 Bu-shen-zhu-yun decoction(BSZY)**								
BSZY-H 2021	77.91	20.15	10	110.15	30.9	5	9.4%	-1.27 [-2.46, -0.07]
BSZY-L 2021	107.46	28.21	10	110.15	30.9	5	10.2%	-0.09 [-1.16, 0.99]
**Subtotal (95% CI)**			**20**			**10**	**19.6%**	**-0.65 [-1.80, 0.51]**
Heterogeneity: Tau² = 0.36; Chi² = 2.06, df = 1 (P = 0.15); I² = 52%								
Test for overall effect: Z = 1.10 (P = 0.27)								
**1.7.6 DXB-2030**								
DXB-2030 2019	57.95	13.41	10	67.49	19	10	11.5%	-0.56 [-1.45, 0.34]
**Subtotal (95% CI)**			**10**			**10**	**11.5%**	**-0.56 [-1.45, 0.34]**
Heterogeneity: Not applicable								
Test for overall effect: Z = 1.21 (P = 0.22)								
**Total (95% CI)**			**118**			**52**	**100.0%**	**-1.01 [-1.58, -0.45]**
Heterogeneity: Tau² = 0.51; Chi² = 25.06, df = 12 (P = 0.01); I² = 52%								
Test for overall effect: Z = 3.52 (P = 0.0004)								
Test for subgroup differences: Chi² = 17.61, df = 5 (P = 0.003), I² = 71.6%								

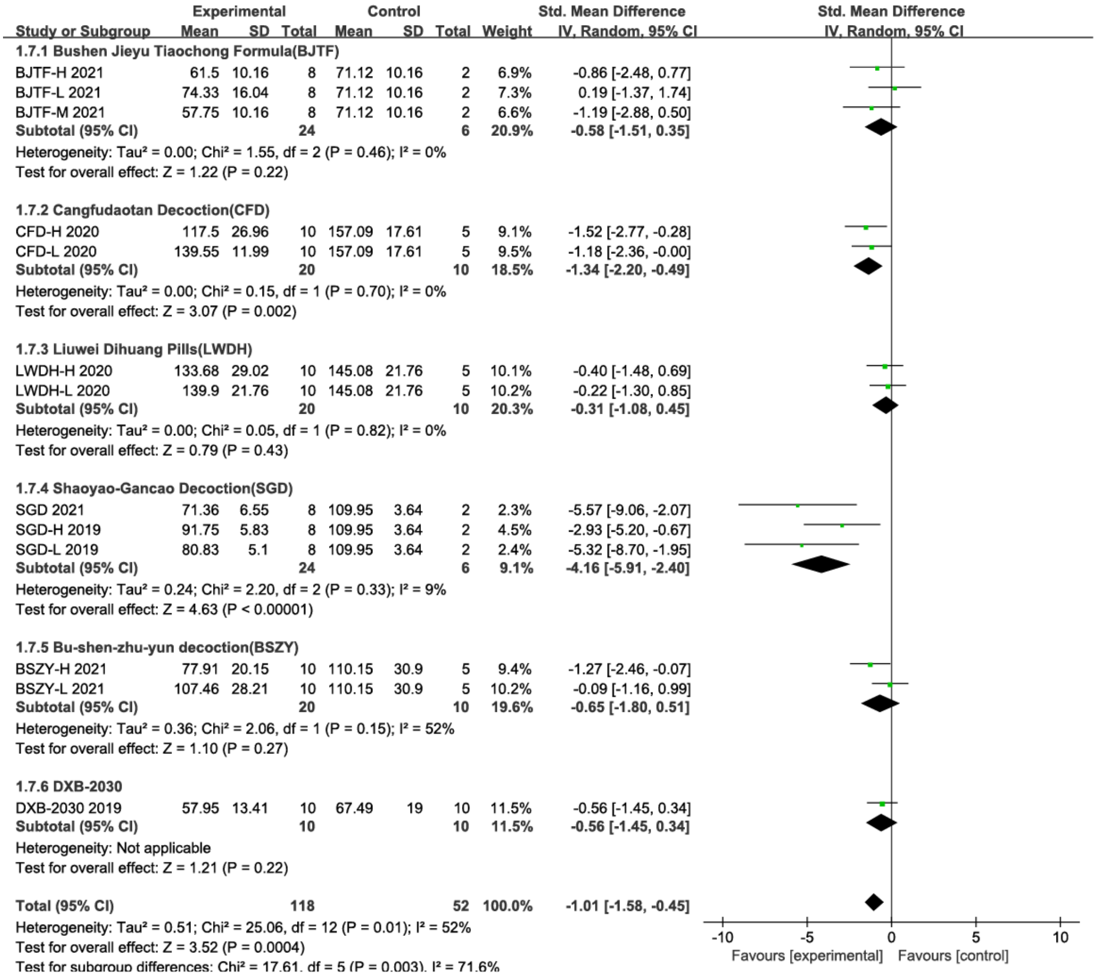

B

Study or Subgroup	Experimental Mean	SD	Total	Control Mean	SD	Total	Weight	Std. Mean Difference IV, Random, 95% CI
**1.22.1 Bushen Jieyu Tiaochong Formula(BJTF)**								
BJTF-H 2021	24.44	6.81	8	27.41	8.3	2	3.6%	-0.38 [-1.95, 1.18]
BJTF-L 2021	23.26	7.41	8	27.41	8.3	2	3.6%	-0.50 [-2.07, 1.08]
BJTF-M 2021	19.41	7.7	8	27.41	8.3	2	3.5%	-0.93 [-2.56, 0.71]
**Subtotal (95% CI)**			**24**			**6**	**10.7%**	**-0.59 [-1.51, 0.32]**
Heterogeneity: Tau² = 0.00; Chi² = 0.25, df = 2 (P = 0.88); I² = 0%								
Test for overall effect: Z = 1.27 (P = 0.20)								
**1.22.2 Shaoyao-Gancao Decoction(SGD)**								
SGD 2021	26.95	8.04	10	34.24	8.84	10	5.1%	-0.83 [-1.75, 0.10]
SGD-H 2019	0.46	0.18	8	0.87	0.25	2	3.0%	-1.95 [-3.85, -0.05]
SGD-L 2019	0.48	0.22	8	0.87	0.25	2	3.2%	-1.57 [-3.36, 0.21]
SGD-M 2019	0.47	0.25	8	0.87	0.25	2	3.3%	-1.45 [-3.20, 0.30]
**Subtotal (95% CI)**			**34**			**16**	**14.6%**	**-1.18 [-1.87, -0.49]**
Heterogeneity: Tau² = 0.00; Chi² = 1.47, df = 3 (P = 0.69); I² = 0%								
Test for overall effect: Z = 3.36 (P = 0.0008)								
**1.22.3 Bushen Huatan Granules(BHG)**								
BHG-H 2021	11.76	11.99	8	56.13	15.59	1	1.8%	-3.29 [-6.19, -0.39]
BHG-L 2021	9.86	10.79	8	56.13	15.59	1	1.6%	-3.81 [-6.95, -0.68]
BHG-M 2021	7.21	10.79	8	56.13	15.59	1	1.5%	-4.03 [-7.27, -0.79]
**Subtotal (95% CI)**			**24**			**3**	**4.8%**	**-3.68 [-5.46, -1.90]**
Heterogeneity: Tau² = 0.00; Chi² = 0.12, df = 2 (P = 0.94); I² = 0%								
Test for overall effect: Z = 4.05 (P < 0.0001)								
**1.22.4 Kunling Wan(KW)**								
KW-H 2021	9.48	9.59	8	56.13	15.59	1	1.4%	-4.32 [-7.70, -0.94]
KW-L 2021	9.48	10.79	8	56.13	15.59	1	1.6%	-3.84 [-6.99, -0.69]
KW-M 2021	7.58	7.2	8	56.13	15.59	1	1.0%	-5.99 [-10.23, -1.76]
**Subtotal (95% CI)**			**24**			**3**	**3.9%**	**-4.51 [-6.53, -2.48]**
Heterogeneity: Tau² = 0.00; Chi² = 0.65, df = 2 (P = 0.72); I² = 0%								
Test for overall effect: Z = 4.36 (P < 0.0001)								
**1.22.5 Guizhi Fuling Wan(GFW)**								
GFW-H 2020	0.76	0.31	12	1.17	0.28	4	4.3%	-1.28 [-2.52, -0.04]
GFW-H 2021	0.75	0.73	12	1.07	0.9	4	4.5%	-0.39 [-1.54, 0.75]
GFW-L 2020	0.79	0.28	12	1.17	0.28	4	4.3%	-1.28 [-2.53, -0.04]
GFW-L 2021	0.78	0.52	12	1.07	0.9	4	4.5%	-0.44 [-1.59, 0.70]
GFW-M 2020	0.76	0.31	12	1.17	0.28	4	4.3%	-1.28 [-2.52, -0.04]
GFW-M 2021	0.76	0.57	12	1.07	0.9	4	4.5%	-0.45 [-1.59, 0.70]
**Subtotal (95% CI)**			**72**			**24**	**26.6%**	**-0.82 [-1.30, -0.33]**
Heterogeneity: Tau² = 0.00; Chi² = 2.93, df = 5 (P = 0.71); I² = 0%								
Test for overall effect: Z = 3.30 (P = 0.0010)								
**1.22.6 Cangfudaotan Decoction(CFD)**								
CFD-H 2020	60.73	6.31	10	68.61	11.04	5	4.6%	-0.92 [-2.06, 0.22]
CFD-L 2020	59.94	7.89	10	68.61	11.04	5	4.6%	-0.91 [-2.05, 0.23]
**Subtotal (95% CI)**			**20**			**10**	**9.1%**	**-0.91 [-1.72, -0.11]**
Heterogeneity: Tau² = 0.00; Chi² = 0.00, df = 1 (P = 0.99); I² = 0%								
Test for overall effect: Z = 2.22 (P = 0.03)								
**1.22.7 FuFang ZhenZhu TiaoZi(FTZ)**								
FTZ 2021	0.21	0.25	6	0.83	0.3	6	3.7%	-2.07 [-3.59, -0.55]
**Subtotal (95% CI)**			**6**			**6**	**3.7%**	**-2.07 [-3.59, -0.55]**
Heterogeneity: Not applicable								
Test for overall effect: Z = 2.68 (P = 0.007)								
**1.22.8 Bao Gui capsule(BGC)**								
BGC-H 2020	12.84	1.4	9	24.87	1.81	4	1.3%	-7.35 [-10.93, -3.77]
BGC-L 2020	14.04	1.2	9	24.87	1.81	4	1.3%	-7.23 [-10.76, -3.70]
**Subtotal (95% CI)**			**18**			**8**	**2.6%**	**-7.29 [-9.80, -4.77]**
Heterogeneity: Tau² = 0.00; Chi² = 0.00, df = 1 (P = 0.96); I² = 0%								
Test for overall effect: Z = 5.68 (P < 0.00001)								
**1.22.9 Liuwei Dihuang Pills(LWDH)**								
LWDH-H 2020	1.42	0.31	10	9.66	3.54	5	2.9%	-3.92 [-5.87, -1.96]
LWDH-L 2020	2.38	0.31	10	2.6	0.35	5	4.6%	-0.64 [-1.75, 0.46]
**Subtotal (95% CI)**			**20**			**10**	**7.6%**	**-2.18 [-5.38, 1.03]**
Heterogeneity: Tau² = 4.71; Chi² = 8.17, df = 1 (P = 0.004); I² = 88%								
Test for overall effect: Z = 1.33 (P = 0.18)								
**1.22.10 DXB-2030**								
DXB-2030 2019	0.05	0.06	10	0.24	0.2	10	4.9%	-1.23 [-2.21, -0.26]
**Subtotal (95% CI)**			**10**			**10**	**4.9%**	**-1.23 [-2.21, -0.26]**
Heterogeneity: Not applicable								
Test for overall effect: Z = 2.48 (P = 0.01)								
**1.22.11 Heqi San(HEQI)**								
HEQI 2017	62.19	23.98	10	95.57	71.95	10	5.1%	-0.60 [-1.50, 0.30]
**Subtotal (95% CI)**			**10**			**10**	**5.1%**	**-0.60 [-1.50, 0.30]**
Heterogeneity: Not applicable								
Test for overall effect: Z = 1.30 (P = 0.19)								
**1.22.12 Shouwu Jiangqi Decoction(SJD)**								
SJD-H 2016	1.85	0.23	8	4.98	0.46	2	0.5%	-10.48 [-16.58, -4.38]
SJD-L 2016	3.95	0.32	9	4.98	0.46	2	2.7%	-2.78 [-4.89, -0.67]
SJD-M 2016	4.11	0.42	9	4.98	0.46	2	3.2%	-1.87 [-3.69, -0.06]
**Subtotal (95% CI)**			**26**			**6**	**6.3%**	**-3.63 [-6.59, -0.67]**
Heterogeneity: Tau² = 4.43; Chi² = 7.05, df = 2 (P = 0.03); I² = 72%								
Test for overall effect: Z = 2.41 (P = 0.02)								
**Total (95% CI)**			**288**			**112**	**100.0%**	**-1.62 [-2.07, -1.16]**
Heterogeneity: Tau² = 0.84; Chi² = 73.11, df = 30 (P < 0.0001); I² = 59%								
Test for overall effect: Z = 6.99 (P < 0.00001)								
Test for subgroup differences: Chi² = 50.70, df = 11 (P < 0.00001), I² = 78.3%								

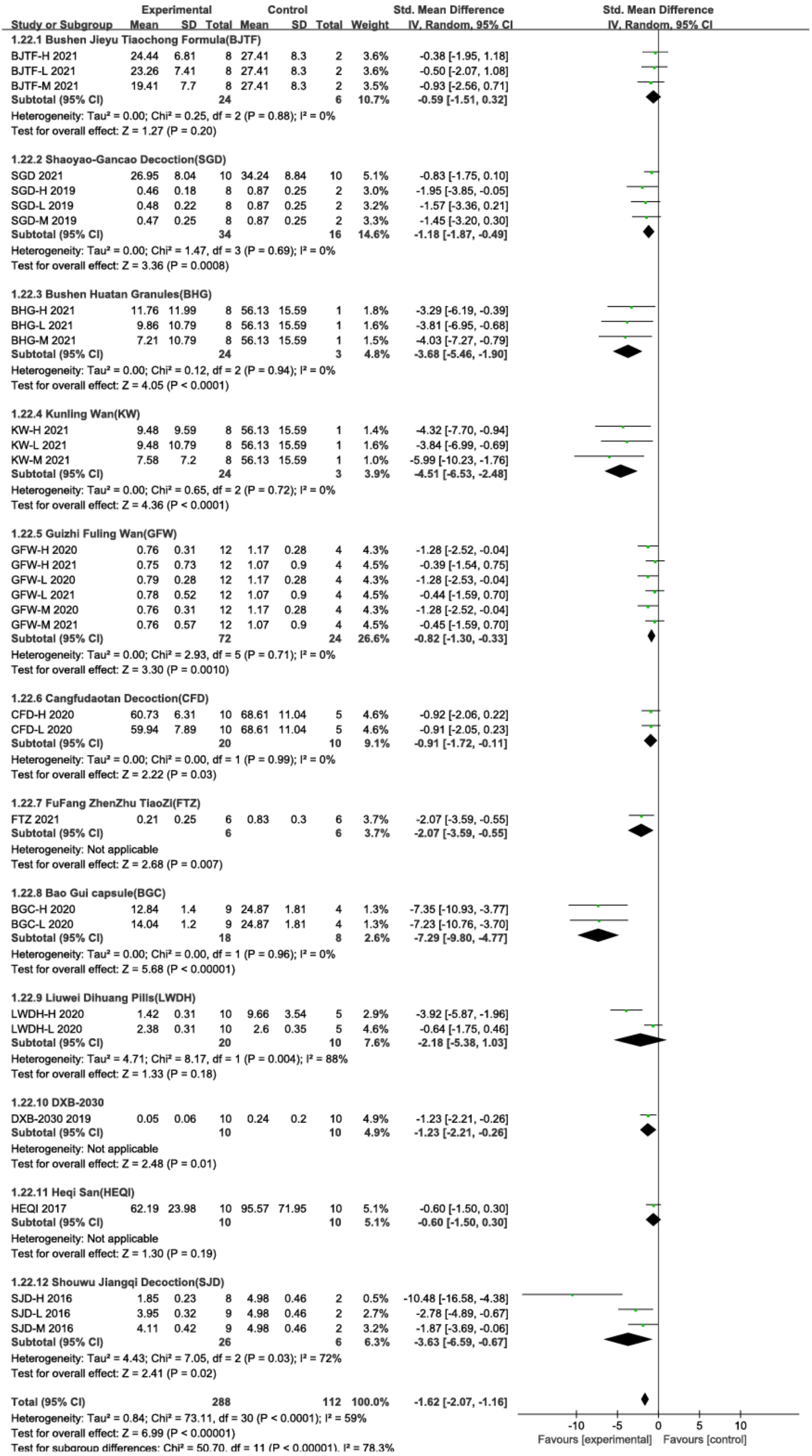

C

Study or Subgroup	Experimental Mean	Experimental SD	Experimental Total	Control Mean	Control SD	Control Total	Weight	Std. Mean Difference IV, Random, 95% CI
**1.2.1 Bushen Jieyu Tiaochong Formula(BJTF)**								
BJTF-H 2021	318.3	25.53	8	335.32	17.02	2	5.5%	-0.62 [-2.21, 0.96]
BJTF-L 2021	320	30.64	8	335.32	17.02	2	5.6%	-0.47 [-2.04, 1.10]
BJTF-M 2021	311.49	22.13	8	335.32	17.02	2	5.1%	-1.00 [-2.65, 0.65]
**Subtotal (95% CI)**			**24**			**6**	**16.2%**	**-0.69 [-1.61, 0.24]**
Heterogeneity: Tau^2 = 0.00; Chi^2 = 0.21, df = 2 (P = 0.90); I^2 = 0%								
Test for overall effect: Z = 1.46 (P = 0.14)								
**1.2.2 Bu-shen-zhu-yun decoction(BSZY)**								
BSZY-H 2021	156.11	2.13	5	168.06	1.71	2	0.8%	-4.90 [-9.12, -0.69]
BSZY-L 2021	160.38	2.56	5	168.06	1.71	2	1.9%	-2.68 [-5.36, 0.00]
**Subtotal (95% CI)**			**10**			**4**	**2.7%**	**-3.32 [-5.58, -1.06]**
Heterogeneity: Tau^2 = 0.00; Chi^2 = 0.76, df = 1 (P = 0.38); I^2 = 0%								
Test for overall effect: Z = 2.88 (P = 0.004)								
**1.2.3 Shaoyao-Gancao Decoction(SGD)**								
SGD 2021	308.98	17.29	10	328.13	23.06	10	16.0%	-0.90 [-1.83, 0.03]
SGD-H 2019	295.56	18	8	329.49	22	2	4.2%	-1.65 [-3.46, 0.15]
SGD-L 2019	306.87	16	8	329.49	22	2	4.8%	-1.21 [-2.90, 0.48]
SGD-M 2019	305.45	14	8	329.49	22	2	4.5%	-1.43 [-3.17, 0.32]
**Subtotal (95% CI)**			**34**			**16**	**29.6%**	**-1.14 [-1.82, -0.46]**
Heterogeneity: Tau^2 = 0.00; Chi^2 = 0.67, df = 3 (P = 0.88); I^2 = 0%								
Test for overall effect: Z = 3.27 (P = 0.001)								
**1.2.4 Bushen Huatan Granules(BHG)**								
BHG-H 2021	241.62	41.28	8	251.35	36.69	1	3.2%	-0.21 [-2.29, 1.87]
BHG-L 2021	238.38	32.11	8	251.35	36.69	1	3.2%	-0.36 [-2.45, 1.73]
BHG-M 2021	244.86	32.11	8	251.35	36.69	1	3.2%	-0.18 [-2.26, 1.90]
**Subtotal (95% CI)**			**24**			**3**	**9.6%**	**-0.25 [-1.45, 0.95]**
Heterogeneity: Tau^2 = 0.00; Chi^2 = 0.02, df = 2 (P = 0.99); I^2 = 0%								
Test for overall effect: Z = 0.41 (P = 0.69)								
**1.2.5 Kunling Wan(KW)**								
KW-H 2021	244.86	32.11	8	251.35	36.69	1	3.2%	-0.18 [-2.26, 1.90]
KW-L 2021	248.11	36.69	8	251.35	36.69	1	3.2%	-0.08 [-2.16, 2.00]
KW-M 2021	251.35	36.69	8	251.35	36.69	1	3.2%	0.00 [-2.08, 2.08]
**Subtotal (95% CI)**			**24**			**3**	**9.6%**	**-0.09 [-1.29, 1.11]**
Heterogeneity: Tau^2 = 0.00; Chi^2 = 0.01, df = 2 (P = 0.99); I^2 = 0%								
Test for overall effect: Z = 0.14 (P = 0.89)								
**1.2.6 Cangfudaotan Decoction(CFD)**								
CFD-H 2020	366.73	12.06	10	393.74	18.1	5	8.1%	-1.79 [-3.10, -0.48]
CFD-L 2020	383.25	18.36	10	393.74	18.1	5	11.5%	-0.54 [-1.64, 0.56]
**Subtotal (95% CI)**			**20**			**10**	**19.6%**	**-1.11 [-2.33, 0.11]**
Heterogeneity: Tau^2 = 0.40; Chi^2 = 2.06, df = 1 (P = 0.15); I^2 = 52%								
Test for overall effect: Z = 1.79 (P = 0.07)								
**1.2.7 Bao Gui capsule(BGC)**								
BGC-H 2020	307.78	22.22	9	342.22	16.67	4	7.3%	-1.54 [-2.91, -0.16]
BGC-L 2020	314.44	7.78	9	342.22	16.67	4	5.4%	-2.36 [-3.96, -0.76]
**Subtotal (95% CI)**			**18**			**8**	**12.7%**	**-1.89 [-2.93, -0.84]**
Heterogeneity: Tau^2 = 0.00; Chi^2 = 0.59, df = 1 (P = 0.44); I^2 = 0%								
Test for overall effect: Z = 3.54 (P = 0.0004)								
**Total (95% CI)**			**154**			**50**	**100.0%**	**-1.02 [-1.39, -0.65]**
Heterogeneity: Tau^2 = 0.00; Chi^2 = 15.45, df = 18 (P = 0.63); I^2 = 0%								
Test for overall effect: Z = 5.36 (P < 0.00001)								
Test for subgroup differences: Chi^2 = 11.14, df = 6 (P = 0.08), I^2 = 46.1%								

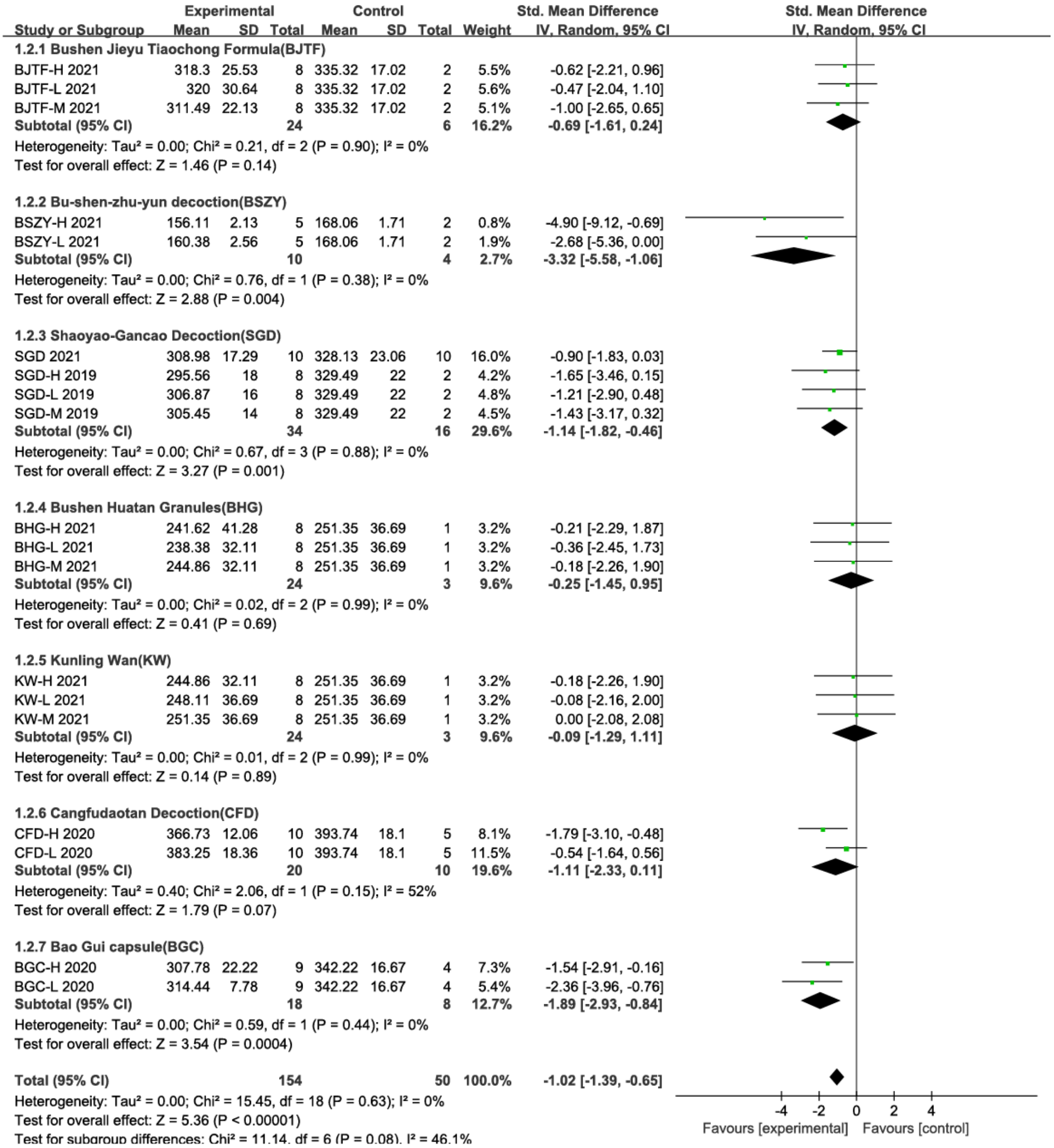

D

Study or Subgroup	Experimental Mean	Experimental SD	Experimental Total	Control Mean	Control SD	Control Total	Weight	Std. Mean Difference IV, Random, 95% CI
**1.12.1 Bushen Jieyu Tiaochong Formula(BJTF)**								
BJTF-H 2021	2.13	0.84	8	2.17	1.36	2	4.4%	-0.04 [-1.59, 1.51]
BJTF-L 2021	1.89	0.86	8	2.17	1.36	2	4.4%	-0.27 [-1.83, 1.29]
BJTF-M 2021	1.87	0.8	8	2.17	1.36	2	4.3%	-0.30 [-1.86, 1.25]
**Subtotal (95% CI)**			**24**			**6**	**13.1%**	**-0.20 [-1.10, 0.69]**
Heterogeneity: Tau² = 0.00; Chi² = 0.07, df = 2 (P = 0.97); I² = 0%								
Test for overall effect: Z = 0.45 (P = 0.66)								
**1.12.2 Shaoyao-Gancao Decoction(SGD)**								
SGD 2021	12.46	10.05	10	9.92	4.82	10	8.1%	0.31 [-0.57, 1.19]
SGD-H 2019	33.33	9.29	8	25.51	2.86	2	4.1%	0.81 [-0.81, 2.42]
SGD-L 2019	30.81	8.57	8	25.51	2.86	2	4.2%	0.59 [-0.99, 2.18]
SGD-M 2019	36.11	5	8	25.51	2.86	2	3.2%	2.00 [0.09, 3.92]
**Subtotal (95% CI)**			**34**			**16**	**19.7%**	**0.64 [-0.02, 1.29]**
Heterogeneity: Tau² = 0.00; Chi² = 2.52, df = 3 (P = 0.47); I² = 0%								
Test for overall effect: Z = 1.91 (P = 0.06)								
**1.12.3 Guizhi Fuling Wan(GFW)**								
GFW-H 2021	29.91	16.24	12	26.34	13.15	4	6.4%	0.22 [-0.92, 1.35]
GFW-L 2021	30.13	30.93	12	26.34	13.15	4	6.4%	0.13 [-1.01, 1.26]
GFW-M 2021	28.35	15.46	12	26.34	13.15	4	6.4%	0.13 [-1.01, 1.26]
**Subtotal (95% CI)**			**36**			**12**	**19.2%**	**0.16 [-0.50, 0.81]**
Heterogeneity: Tau² = 0.00; Chi² = 0.02, df = 2 (P = 0.99); I² = 0%								
Test for overall effect: Z = 0.47 (P = 0.64)								
**1.12.4 Cangfudaotan Decoction(CFD)**								
CFD-H 2020	9	1.09	10	9.14	1.23	5	6.7%	-0.12 [-1.19, 0.96]
CFD-H 2021	9.11	0.27	6	5.09	1.07	3	1.0%	5.80 [1.97, 9.64]
CFD-L 2020	9.55	1.36	10	9.14	1.23	5	6.7%	0.29 [-0.79, 1.37]
CFD-L 2021	6.43	0.8	6	5.09	1.07	3	4.1%	1.35 [-0.27, 2.96]
**Subtotal (95% CI)**			**32**			**16**	**18.6%**	**0.97 [-0.41, 2.36]**
Heterogeneity: Tau² = 1.25; Chi² = 9.80, df = 3 (P = 0.02); I² = 69%								
Test for overall effect: Z = 1.38 (P = 0.17)								
**1.12.5 Liuwei Dihuang Pills(LWDH)**								
LWDH-H 2020	1.72	0.21	10	1.03	0.23	5	4.0%	3.00 [1.35, 4.65]
LWDH-L 2020	1.41	0.34	10	1.03	0.23	5	6.1%	1.15 [-0.02, 2.33]
**Subtotal (95% CI)**			**20**			**10**	**10.2%**	**1.98 [0.18, 3.79]**
Heterogeneity: Tau² = 1.18; Chi² = 3.20, df = 1 (P = 0.07); I² = 69%								
Test for overall effect: Z = 2.16 (P = 0.03)								
**1.12.6 Heqi San(HEQI)**								
HEQI 2017	10.41	4.15	10	10	6.22	10	8.1%	0.07 [-0.80, 0.95]
**Subtotal (95% CI)**			**10**			**10**	**8.1%**	**0.07 [-0.80, 0.95]**
Heterogeneity: Not applicable								
Test for overall effect: Z = 0.17 (P = 0.87)								
**1.12.7 Shouwu Jiangqi Decoction(SJD)**								
SJD-H 2016	3.02	0.25	8	2.13	0.42	2	2.5%	2.90 [0.65, 5.15]
SJD-L 2016	2.21	0.29	9	2.13	0.42	2	4.4%	0.24 [-1.30, 1.78]
SJD-M 2016	2.34	0.35	9	2.13	0.42	2	4.3%	0.54 [-1.02, 2.09]
**Subtotal (95% CI)**			**26**			**6**	**11.3%**	**1.02 [-0.40, 2.43]**
Heterogeneity: Tau² = 0.77; Chi² = 3.95, df = 2 (P = 0.14); I² = 49%								
Test for overall effect: Z = 1.40 (P = 0.16)								
**Total (95% CI)**			**182**			**76**	**100.0%**	**0.58 [0.19, 0.97]**
Heterogeneity: Tau² = 0.30; Chi² = 31.41, df = 19 (P = 0.04); I² = 40%								
Test for overall effect: Z = 2.88 (P = 0.004)								
Test for subgroup differences: Chi² = 7.64, df = 6 (P = 0.27), I² = 21.5%								

Std. Mean Difference
IV, Random, 95% CI

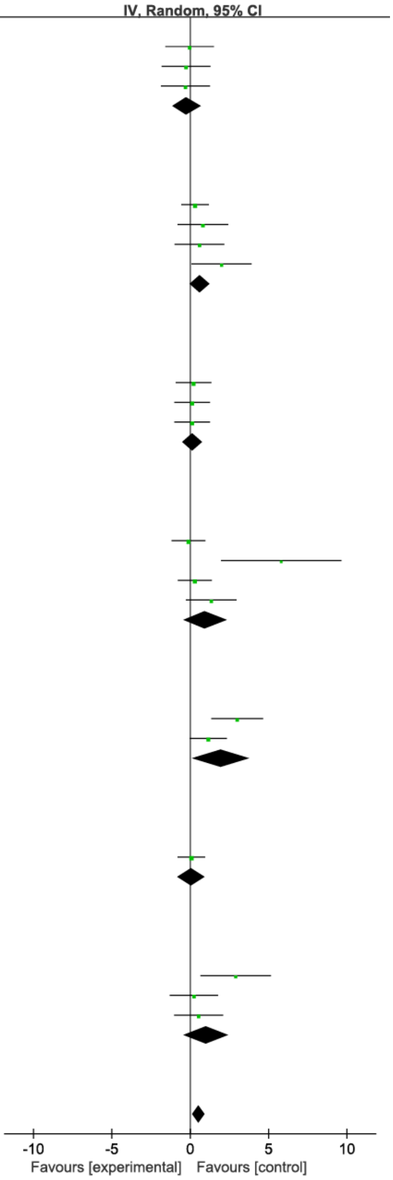

E

Study or Subgroup	Experimental Mean	Experimental SD	Experimental Total	Control Mean	Control SD	Control Total	Weight	Std. Mean Difference IV, Random, 95% CI
**1.17.1 Bushen Jieyu Tiaochong Formula(BJTF)**								
BJTF-H 2021	0.53	0.52	8	1.19	0.49	2	3.2%	-1.15 [-2.83, 0.53]
BJTF-L 2021	0.84	0.36	8	1.19	0.49	2	3.4%	-0.83 [-2.45, 0.78]
BJTF-M 2021	0.43	0.21	8	1.19	0.49	2	2.0%	-2.62 [-4.76, -0.48]
**Subtotal (95% CI)**			**24**			**6**	**8.6%**	**-1.36 [-2.39, -0.34]**
Heterogeneity: Tau² = 0.00; Chi² = 1.80, df = 2 (P = 0.41); I² = 0%								
Test for overall effect: Z = 2.61 (P = 0.009)								
**1.17.2 Shaoyao-Gancao Decoction(SGD)**								
SGD 2021	42.78	17.61	10	50.89	12.01	10	10.2%	-0.52 [-1.41, 0.38]
SGD-H 2019	77.25	2.12	8	84	19.09	2	3.4%	-0.87 [-2.49, 0.76]
SGD-L 2019	75.75	16.97	8	84	19.09	2	3.6%	-0.43 [-2.00, 1.14]
SGD-M 2019	79.5	2.12	8	84	19.09	2	3.6%	-0.58 [-2.16, 1.01]
**Subtotal (95% CI)**			**34**			**16**	**20.9%**	**-0.57 [-1.21, 0.07]**
Heterogeneity: Tau² = 0.00; Chi² = 0.17, df = 3 (P = 0.98); I² = 0%								
Test for overall effect: Z = 1.73 (P = 0.08)								
**1.17.3 Guizhi Fuling Wan(GFW)**								
GFW-H 2021	41.59	18.31	12	54.98	39.07	4	6.5%	-0.52 [-1.67, 0.63]
GFW-L 2021	38.06	13.43	12	54.98	39.07	4	6.3%	-0.74 [-1.91, 0.43]
GFW-M 2021	41.59	21.97	12	54.98	39.07	4	6.6%	-0.48 [-1.62, 0.67]
**Subtotal (95% CI)**			**36**			**12**	**19.4%**	**-0.58 [-1.24, 0.09]**
Heterogeneity: Tau² = 0.00; Chi² = 0.11, df = 2 (P = 0.95); I² = 0%								
Test for overall effect: Z = 1.69 (P = 0.09)								
**1.17.4 Cangfudaotan Decoction(CFD)**								
CFD-H 2020	27.73	6.36	10	37.27	6.82	5	5.9%	-1.38 [-2.60, -0.16]
CFD-H 2021	3.48	1.07	6	10.71	1.61	3	0.8%	-5.15 [-8.61, -1.69]
CFD-L 2020	31.82	3.64	10	37.27	6.82	5	6.4%	-1.06 [-2.22, 0.10]
CFD-L 2021	6.43	1.34	6	10.71	1.61	3	2.0%	-2.67 [-4.83, -0.52]
**Subtotal (95% CI)**			**32**			**16**	**15.0%**	**-1.89 [-3.09, -0.69]**
Heterogeneity: Tau² = 0.70; Chi² = 5.95, df = 3 (P = 0.11); I² = 50%								
Test for overall effect: Z = 3.08 (P = 0.002)								
**1.17.5 FuFang ZhenZhu TiaoZi(FTZ)**								
FTZ 2021	4.75	0.6	6	7.25	1.92	6	4.6%	-1.62 [-3.00, -0.24]
**Subtotal (95% CI)**			**6**			**6**	**4.6%**	**-1.62 [-3.00, -0.24]**
Heterogeneity: Not applicable								
Test for overall effect: Z = 2.30 (P = 0.02)								
**1.17.6 Liuwei Dihuang Pills(LWDH)**								
LWDH-H 2020	4.47	1.3	10	7.33	1.55	5	4.9%	-1.95 [-3.29, -0.60]
LWDH-L 2020	5.47	0.87	10	7.33	1.55	5	5.6%	-1.56 [-2.81, -0.30]
**Subtotal (95% CI)**			**20**			**10**	**10.4%**	**-1.74 [-2.66, -0.82]**
Heterogeneity: Tau² = 0.00; Chi² = 0.17, df = 1 (P = 0.68); I² = 0%								
Test for overall effect: Z = 3.71 (P = 0.0002)								
**1.17.7 Heqi San(HEQI)**								
HEQI 2017	25.41	9.5	10	35.52	12.96	10	9.6%	-0.85 [-1.78, 0.07]
**Subtotal (95% CI)**			**10**			**10**	**9.6%**	**-0.85 [-1.78, 0.07]**
Heterogeneity: Not applicable								
Test for overall effect: Z = 1.81 (P = 0.07)								
**1.17.8 Shouwu Jiangqi Decoction(SJD)**								
SJD-H 2016	8.22	1.78	8	8.43	1.43	2	3.7%	-0.11 [-1.66, 1.44]
SJD-L 2016	8.19	1.27	9	8.43	1.43	2	3.8%	-0.17 [-1.71, 1.36]
SJD-M 2016	8.35	1.32	9	8.43	1.43	2	3.8%	-0.05 [-1.59, 1.48]
**Subtotal (95% CI)**			**26**			**6**	**11.3%**	**-0.11 [-1.00, 0.78]**
Heterogeneity: Tau² = 0.00; Chi² = 0.01, df = 2 (P = 0.99); I² = 0%								
Test for overall effect: Z = 0.25 (P = 0.81)								
**Total (95% CI)**			**188**			**82**	**100.0%**	**-0.94 [-1.25, -0.64]**
Heterogeneity: Tau² = 0.03; Chi² = 21.30, df = 20 (P = 0.38); I² = 6%								
Test for overall effect: Z = 6.02 (P < 0.00001)								
Test for subgroup differences: Chi² = 12.63, df = 7 (P = 0.08), I² = 44.6%								

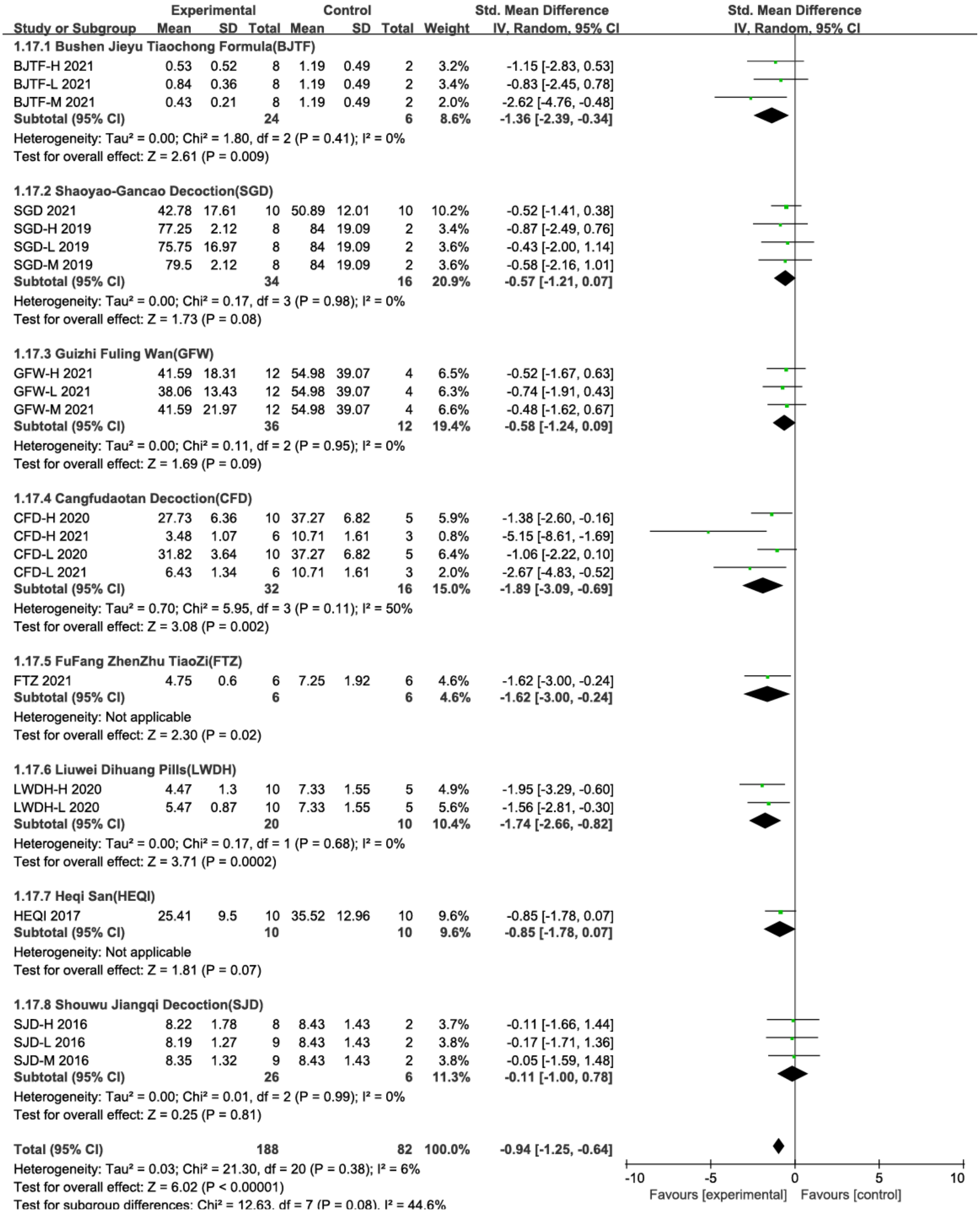

F

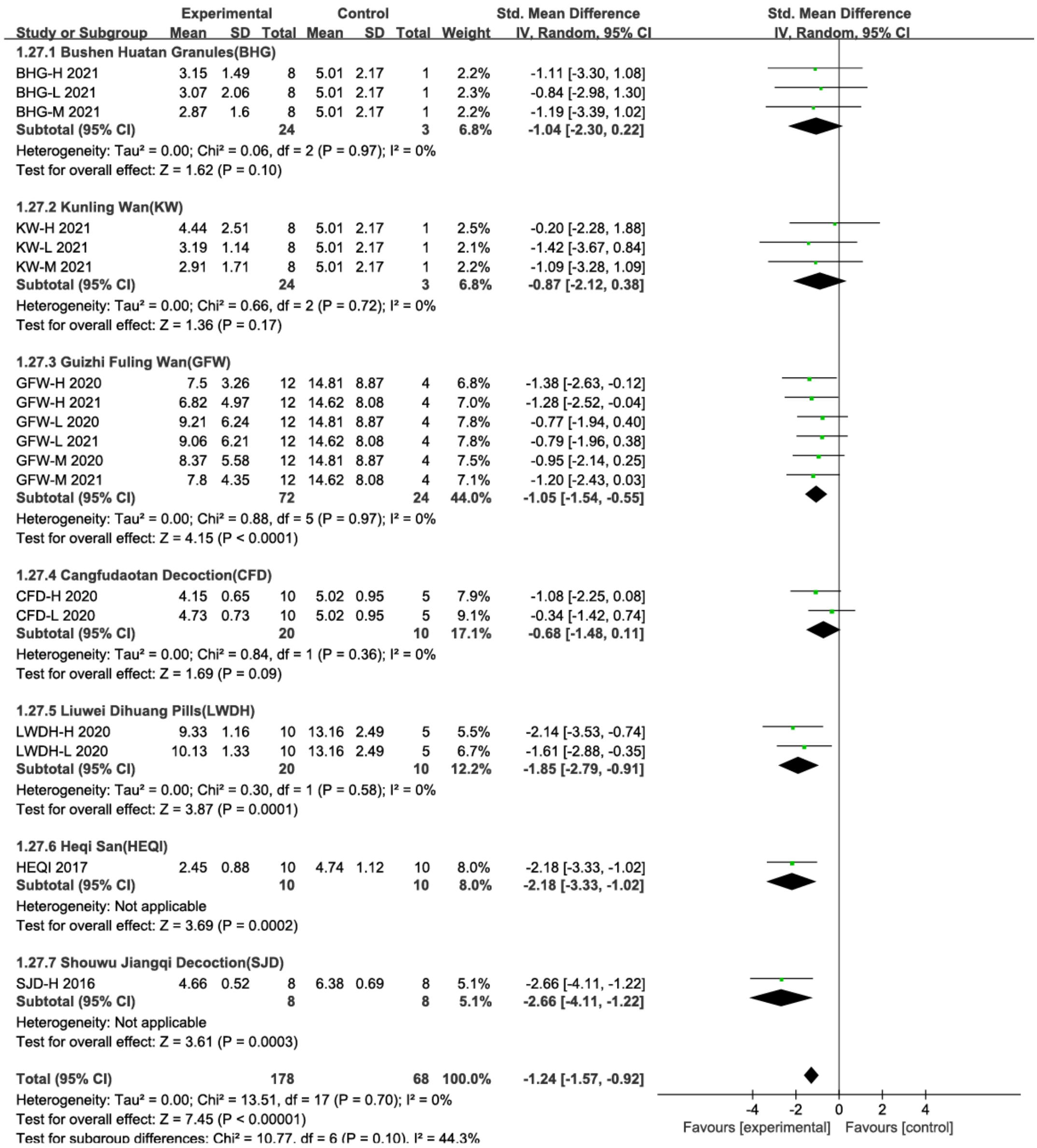

Supplementary Fig. 3 According to different formulas, meta-analysis was conducted for (A) ovarian mass, (B) testosterone, (C) weight, (D) FSH, (E) LH, and(F) HOMA-IR.

Supplementary Fig. 4 Sensitivity analysis

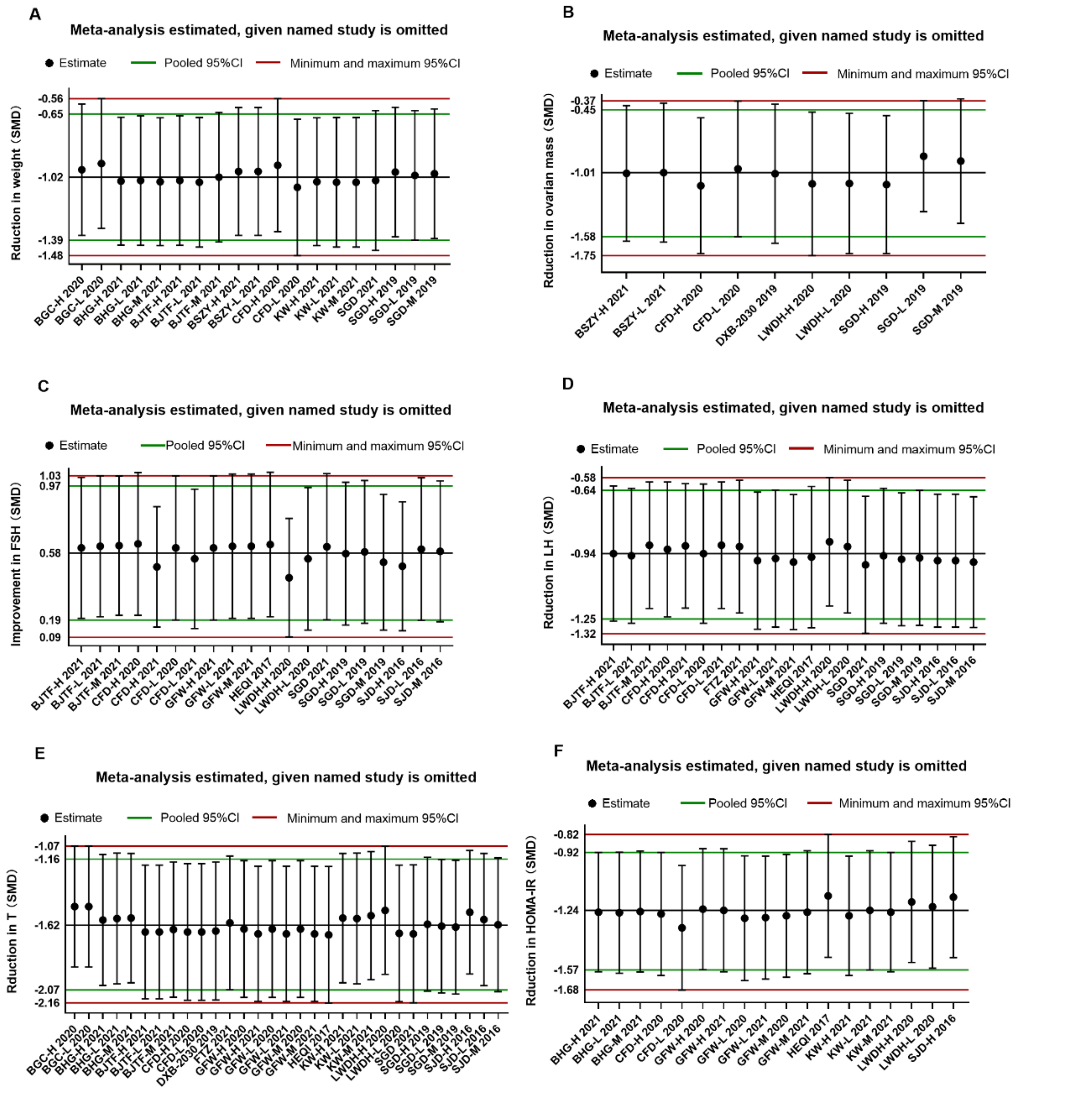

Supplementary Fig. 4 The sensitivity analysis was conducted for (A) weight, (B) ovarian mass, (C) FSH, (D) LH, (E) testosterone, and (F) HOMA-IR.

Supplementary Fig. 5 Forest plots of weight improvement by different PCOS induction drugs

Study or Subgroup	Experimental Mean	Experimental SD	Experimental Total	Control Mean	Control SD	Control Total	Weight	Std. Mean Difference IV, Random, 95% CI
**1.1.1 Letrozole**								
BGC-H 2020	307.78	22.22	9	342.22	16.67	4	7.3%	-1.54 [-2.91, -0.16]
BGC-L 2020	314.44	7.78	9	342.22	16.67	4	5.4%	-2.36 [-3.96, -0.76]
BJTF-H 2021	318.3	25.53	8	335.32	17.02	2	5.5%	-0.62 [-2.21, 0.96]
BJTF-L 2021	320	30.64	8	335.32	17.02	2	5.6%	-0.47 [-2.04, 1.10]
BJTF-M 2021	311.49	22.13	8	335.32	17.02	2	5.1%	-1.00 [-2.65, 0.65]
CFD-H 2020	366.73	12.06	10	393.74	18.1	5	8.1%	-1.79 [-3.10, -0.48]
CFD-L 2020	383.25	18.36	10	393.74	18.1	5	11.5%	-0.54 [-1.64, 0.56]
SGD 2021	308.98	17.29	10	328.13	23.06	10	16.0%	-0.90 [-1.83, 0.03]
SGD-H 2019	295.56	18	8	329.49	22	2	4.2%	-1.65 [-3.46, 0.15]
SGD-L 2019	306.87	16	8	329.49	22	2	4.8%	-1.21 [-2.90, 0.48]
SGD-M 2019	305.45	14	8	329.49	22	2	4.5%	-1.43 [-3.17, 0.32]
**Subtotal (95% CI)**			**96**			**40**	**78.1%**	**-1.15 [-1.57, -0.73]**
Heterogeneity: Tau² = 0.00; Chi² = 6.45, df = 10 (P = 0.78); I² = 0%								
Test for overall effect: Z = 5.34 (P < 0.00001)								
**1.1.2 DHEA**								
BHG-H 2021	241.62	41.28	8	251.35	36.69	1	3.2%	-0.21 [-2.29, 1.87]
BHG-L 2021	238.38	32.11	8	251.35	36.69	1	3.2%	-0.36 [-2.45, 1.73]
BHG-M 2021	244.86	32.11	8	251.35	36.69	1	3.2%	-0.18 [-2.26, 1.90]
BSZY-H 2021	156.11	2.13	5	168.06	1.71	2	0.8%	-4.90 [-9.12, -0.69]
BSZY-L 2021	160.38	2.56	5	168.06	1.71	2	1.9%	-2.68 [-5.36, 0.00]
KW-H 2021	244.86	32.11	8	251.35	36.69	1	3.2%	-0.18 [-2.26, 1.90]
KW-L 2021	248.11	36.69	8	251.35	36.69	1	3.2%	-0.08 [-2.16, 2.00]
KW-M 2021	251.35	36.69	8	251.35	36.69	1	3.2%	0.00 [-2.08, 2.08]
**Subtotal (95% CI)**			**58**			**10**	**21.9%**	**-0.57 [-1.39, 0.25]**
Heterogeneity: Tau² = 0.07; Chi² = 7.36, df = 7 (P = 0.39); I² = 5%								
Test for overall effect: Z = 1.36 (P = 0.17)								
**Total (95% CI)**			**154**			**50**	**100.0%**	**-1.02 [-1.39, -0.65]**
Heterogeneity: Tau² = 0.00; Chi² = 15.45, df = 18 (P = 0.63); I² = 0%								
Test for overall effect: Z = 5.36 (P < 0.00001)								
Test for subgroup differences: Chi² = 1.52, df = 1 (P = 0.22), I² = 34.1%								

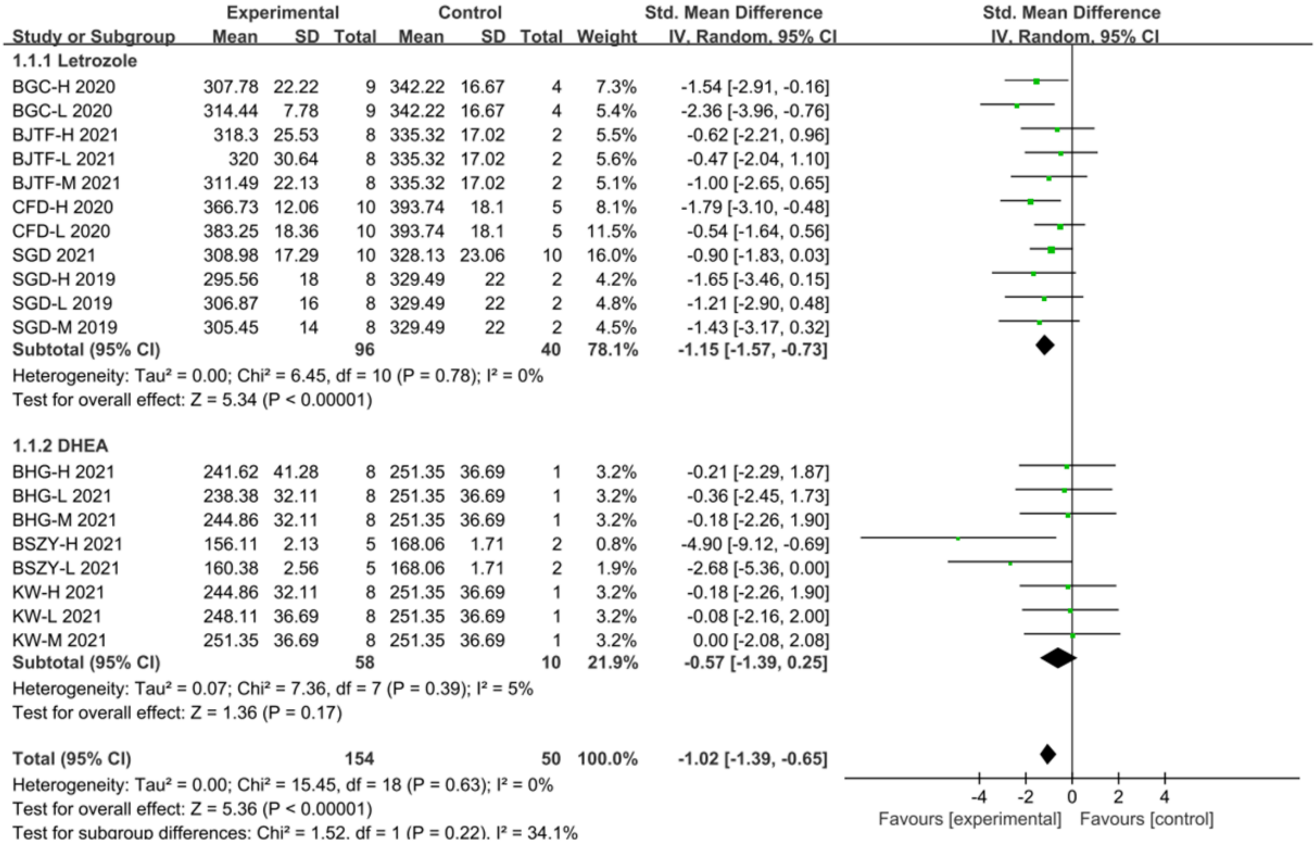

Supplement: Supplementary Materials — Supplementary File 1: Search strategy. Table S1: Study quality score report. Figure S1: Forest plot of the efficacy of CHM on ovarian mass. Figure S2: Forest plot of the efficacy of CHM on testosterone. Figure S3: Forest plots of the efficacy of different formulas. Figure S4: Sensitivity analysis. Figure S5: Forest plots of weight improvement by different PCOS induction drugs. [file 4892215.f1.zip › 4892215.f1/SUPPLEMENTAL MATERIAL (1).pdf]
